# Dynamic patterns of blood lipids and DNA methylation in response to statin therapy

**DOI:** 10.1186/s13148-022-01375-8

**Published:** 2022-11-28

**Authors:** Xueying Qin, Yunzhang Wang, Nancy L. Pedersen, Bowen Tang, Sara Hägg

**Affiliations:** 1 Department of Epidemiology and Biostatistics, School of Public Health, Peking University, 38# Xueyuan Road, Beijing, 100191 China; 2 Department of Medical Epidemiology and Biostatistics, Karolinska Institutet, Nobels Väg 12A, 17177 Stockholm, Sweden

**Keywords:** DNA methylation, Blood lipids, Statin treatment

## Abstract

**Introduction:**

Statins are lipid-lowering drugs and starting treatment has been associated with DNA methylation changes at genes related to lipid metabolism. However, the longitudinal pattern of how statins affect DNA methylation in relation to lipid levels has not been well investigated.

**Methods:**

We conducted an epigenetic association study in a longitudinal Swedish twin sample in previously reported lipid-related CpGs (cg10177197, cg17901584 and cg27243685). First, we applied a mixed-effect model to assess the association between blood lipids (total cholesterol (TC), low-density lipoprotein cholesterol (LDL), high-density lipoprotein cholesterol (HDL), total triglyceride (TG)) and DNA methylation. Then, we performed a piecewise latent linear–linear growth curve model (LGCM) to explore the long-term changing pattern of lipids and methylation in response to statin treatment. Finally, we used a bivariate autoregressive latent trajectory model with structured residuals (ALT-SR) to analyze the cross-lagged effects in different lipid-CpG pairs in statin users and non-users.

**Results:**

We replicated the associations between TC, LDL, HDL and DNA methylation level in cg17901584 and cg27243685 (*P* values ranged from 4.70E−12 to 1.84E−04). From the piecewise LGCM, we showed that TC and LDL significantly decreased in statin users before treatment started and then remained stable. For non-statin users, we only found a slightly significant decreasing trend for TC and TG. We observed a similar dynamic pattern for methylation levels at cg27243685 and cg17901584. Before statin initiation, cg27243685 showed a significantly increasing trend and cg17901584 a decreasing trend, but post-treatment, there were no additional changes. From the ALT-SR model, we found TG levels to be significantly associated with the DNA methylation level of cg27243685 at the next measurement in statin users (estimate = 0.383, 95% CI: 0.173, 0.594, *P* value < 0.001).

**Conclusions:**

Longitudinal blood lipid and DNA methylation levels change after statin treatment initiation, where the latter is mostly a response to alterations in lipid levels and not vice versa.

**Supplementary information:**

The online version contains supplementary material available at 10.1186/s13148-022-01375-8.

## Introduction

DNA methylation is an epigenetic mechanism that involves the addition of a methyl group to the 5’-position of a cytosine and regulates gene expression without changing the DNA sequence. DNA methylation is commonly found in gene promoters containing numerous cytosine followed by guanine dinucleotides (CpGs). The process can be a response to environmental and lifestyle exposures, and may be involved in the mechanisms contributing to disease, such as cardiovascular disease (CVD) [1].

Elevated levels of blood lipids are important risk factors for CVD. Previous epigenome-wide association studies (EWAS) showed an association between elevated blood lipids and methylation at CpGs in genes involved in lipid metabolism, such as the 24-dehydrocholesterol reductase (*DHCR24*) and ATP-binding cassette member-1 subfamily G (*ABCG1*) genes [2–4]. Statins are drugs that effectively lower circulating lipid levels and protect against CVD. A prior epigenetic study showed that statin use is associated with DNA methylation levels at certain CpGs in the genome, particularly in the above-mentioned genes [5]. However, the longitudinal pattern of DNA methylation changes in response to statin therapy has not been clarified, particularly the direction of effects, if lipid changes come first, or if methylation changes come first under the statin treatment.

**Fig. 1:**
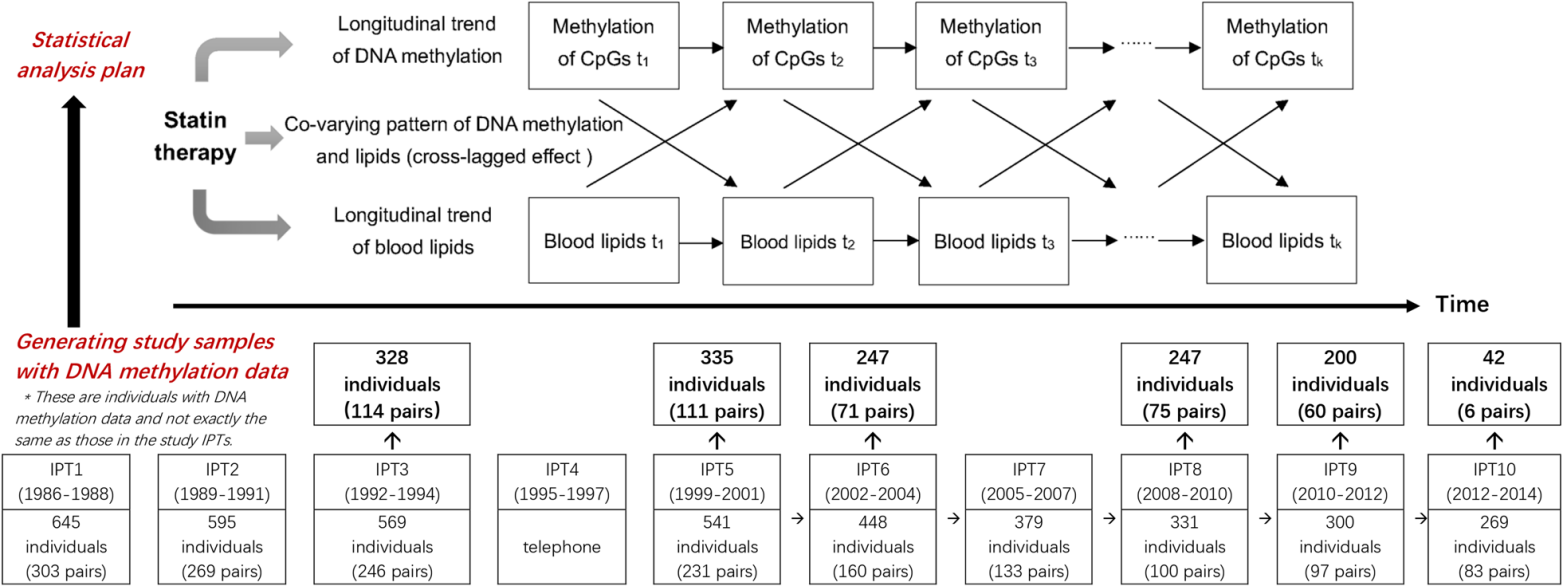
The scheme of study design. This figure describes how the study sample generated (below part) and the statistical analysis plan (up part). The study sample is a sub-sample of SATSA population who participated in in-person testing (IPT) from the first IPT (IPT1, 1986–1988) to the last one (IPT10, 2012–2014). Participants in IPT1 were those who responded to questionnaire 1 and were above 50 years old at the start of IPT1. From IPT2 to IPT5, participants included those who had taken part in the previous IPT and new twins who had recently turned 50 years old. From IPT6 and onwards, there were no new twins added, only longitudinal followed-up (Arrows from IPT5 to IPT10 represent only following-up and no new twins added). DNA methylation was measured in participants attending at least one of these IPTs (IPT3, IPT5, IPT6, IPT8, IPT9, IPT10). Based on the samples with DNA measurements in SATSA, we conducted a CpG-based epigenetic association study in a longitudinal Swedish twin sample to test the effect of statin treatment on the longitudinal trend of blood lipids and on the longitudinal changes of DNA methylation in selected CpGs, and to explore the long-term co-varying pattern of blood lipids and DNA methylation levels of these CpGs in response to statin therapy. SATSA: the Swedish Adoption/Twin Study of Aging; IPT: in-person testing

Here, we conducted a CpG-based epigenetic association study in a longitudinal Swedish twin sample to (1) replicate the association between blood lipids and DNA methylation of candidate CpGs, (2) evaluate the effect of statin treatment on the longitudinal trend of blood lipids, (3) examine the influence of statin treatment on DNA methylation changes in selected CpGs, and (4) explore the co-varying dynamic pattern over time of blood lipids and DNA methylation levels of these CpGs in response to statin therapy. The scheme of the study design is shown in Fig. 1.

## Methods

### Study population

The study population included individuals from the Swedish Adoption/Twin Study of Aging (SATSA), a sub-study of the Swedish Twin Registry (STR). Details on the study design, sample characteristics, and methods of data collection have been described in previous publications [6–8]. Briefly, SATSA, launched in 1984, has overall 2018 participants and includes 10 waves of repeated measurements of various aspects of aging-related information from Swedish twin pairs [6]. In each wave, the individuals were examined using a comprehensive questionnaire survey to collect participants’ information concerning their working environment and health-related behaviors. In addition, in-person testing (IPT) was performed with health examinations, structured cognitive tests, and blood sample collection in a subsample of the SATSA cohort [9]. The naming convention was “IPT” with a number (for example, first IPT was IPT1). IPT1 was performed in 1986 to 1988, and then every third year, a new IPT was conducted. After IPT8 the interval was every second year. A total of ten IPTs were conducted through 2014. IPT4 was excluded because it was implemented only through telephone survey. For the inclusion and exclusion of IPT samples, briefly, participants in IPT1 were those who responded to questionnaire 1 and were above 50 years old at the start of IPT1. From IPT2 to IPT5, participants included those who had taken part in the previous IPT and new twins who had recently turned 50 years old. From IPT6 and onwards, there were no new twins added, only longitudinal followed-up (Fig. 1).

Totally, 861 individuals have participated in at least one wave of IPT in the SATSA cohort. DNA methylation was measured in 536 individuals attending at least one of these IPTs (IPT3, IPT5, IPT6, IPT8, IPT9, IPT10). After excluding one individual’s samples used for quality control of different DNA methylation chip assays, 535 individuals corresponding to 1399 samples with up to six DNA methylation measurements were included in the present study (Fig. 1). Of 535 participants, there were 83 MZ twin pairs, 155 DZ twin pairs and 59 singletons. Singleton was defined as those individuals in the cohort who only have his/her own information but we do not have information about his/her twin. All participants provided informed consent, and ethics approval for this study was given by the Regional Ethics Board at Karolinska Institutet, Stockholm, Sweden.

### Definition and measurement of lipids and statin use

Serum was extracted from whole blood and stored at − 70 °C for the estimation of lipid profiles. Total triglyceride (TG) and total cholesterol (TC) levels were measured by the enzymatic calorimetric method at all IPTs except IPT4. High-density lipoprotein cholesterol (HDL) was measured by the precipitation method at IPT1 and homogeneous assays were used in later IPTs [10]. Outliers were removed as described in a previous study [11]. Low-density lipoprotein cholesterol (LDL) was tested only at IPT7-10; therefore, we used the Friedewald equation [12] “LDL = TC—HDL—TG/5” to impute the missing values of LDL. The correlation between LDL measured and LDL calculated was 98.8%, demonstrating the high quality of imputation. In general, Friedewald equation is not valid for TG levels greater than 400 mg/dL (equal to 4.52 by multiplying by 0.0113 to convert to millimoles per liter (mmol/L)) and direct measurement of LDL is preferred in this situation. We have 1.5% (21/1399) of samples with TG higher than 400 mg/dL; however, their direct LDL measurements were missing, we hence still chose to use calculated LDL for TG levels higher than 400 mg/dL, but sensitivity analyses were further performed by excluding those 21 participants. All lipid values are expressed in mmol/L.

Information on statin use was obtained from self-reported records in IPTs and from data linked from the Swedish Prescribed Drug Register available from 2005 and onwards. In our dataset, the most commonly used statin was simvastatin, which accounted for approximately 77% of all statin use. Atorvastatin is the second commonly used statin, followed by Rosuvastatin, Pravastatin and Fluvastatin. We defined “statin users” as individuals with at least one record of taking any types of statin therapy and coded it as 1. Likewise, “non-statin users” were coded as 0.

### DNA methylation measurements

Blood samples for DNA methylation testing were collected from a subsample of SATSA participants [7, 8]. DNA was extracted from peripheral blood and then treated with bisulfite using the EZ-96 DNA MagPrep methylation kit (Zymo Research, Irvine & Tustin, California, USA). Using bisulfite-converted DNA, methylation of DNA was quantified by the Infinium HumanMethylation450 BeadChip (Illumina, Inc., San Diego, California, USA) or Infinium Methylation EPIC BeadChip assays (Illumina, Inc.). Methylation data were harmonized using a preprocessing pipeline including quality control, normalization and adjustment for cell counts and batch effects, as described in previous studies [7, 8]. Finally, 255,356 CpGs from both arrays remained in the analysis, and repeated measurements of DNA methylation were distributed over IPT3, IPT5, IPT6, IPT8, IPT9, and IPT10. Beta values ranging between 0 and 1 were used as DNA methylation levels.

For the selection of statin-associated CpGs, five candidate CpGs were chosen from Ochoa-Rosales et al. [5]. The first two, cg17901584 and cg10177197, are located in the *DHCR24* gene that encodes an enzyme involved in cholesterol biosynthesis, oxidative stress response, neuroprotection, anti-apoptosis and anti-inflammatory activities [13]. The other two, cg06500161 and cg27243685, are located in the *ABCG1* gene that encodes a protein that plays an important role in the process of reverse cholesterol transport [14]. The last one, cg05119988, is located in sterol-C4-methyl oxidase–like gene that encodes enzyme to catalyze the demethylation of C4-methylsterols in the cholesterol synthesis pathway. However, cg06500161 and cg05119988 were excluded from our analysis because they failed to pass the quality control procedure.

### Statistical analysis

To replicate the longitudinal association between blood lipids and DNA methylation in selected CpGs (aim 1), we used a linear mixed effect model. DNA methylation level was used as a dependent variable and lipid level as an independent variable. Age (repeated measurement, continuous variable), sex (binary variable, women/men coded as 1/0), and smoking (repeated measurement; non-smoker, ex-smoker and current smoker coded as 1, 2 and 3, respectively) were covariates. Individual ID nested within twin pair ID was entered as a random effect, other variables were fixed effects.

For aim 2 and 3, examining the statin effect on the longitudinal changes of blood lipids and DNA methylation, a piecewise latent linear–linear growth curve model (LGCM) was used. Details on the model are presented in Additional file 1: Methods, Figure S1, and Tables S1-S2. Briefly, the piecewise LGCM was constructed in the framework of a structural equation model (SEM), and in our study it was composed of two separate linear segments that were connected by a knot (or change point), here defined as the IPT of starting statin use. The piecewise LGCM has three latent variables: one intercept measuring the individual lipid (or methylation) level at the knot and two slopes (denoted as Pre_slope and Post_slope, respectively) measuring the changing rate of lipids (or DNA methylation) over time before and after statin therapy for each individual. The piecewise LGCM was established separately for statin users and non-users. Of note, non-statin users did not have a change point and the piecewise LGCM was reduced to one phase with only two latent variables (intercept and Pre_slope). We included sex, baseline age, and statin use as time-independent covariates in the regression equation of latent intercepts and slopes, as we assumed that these covariates would have associations with these latent variables [5, 7, 8, 15]. However, statin use only had associations with Pre_slopes.

To fulfill aim 4, to examine the co-varying dynamic patterns of blood lipids and DNA methylation levels across multiple time points in response to statin treatment, we used a bivariate autoregressive latent trajectory model with structured residuals (ALT-SR model) stratified by status of statin therapy. We used the same ALT-SR model as in our previous research [16]. More details are found in Additional file 1: Method and Figure S2. Briefly, the ALT-SR model is also established in the frame of SEM and composed of two parts: the autoregressive model (AR) and the linear latent growth curve model (LGM). The LGM was established first by the latent intercept and the latent slope, which had the same interpretation as in the piecewise LGCM. The AR model measured the co-varying pattern of DNA methylation and lipids over time and was then established based on two regression paths: the autoregressive path measuring the within-person changes of DNA methylation or lipids over time, and the cross-lagged path measuring the predicted effect of DNA methylation at one time point on the within-person lipid level at the adjacent later time point, and/or vice versa. The ALT-SR was constructed separately in statin users and non-users, and differences between the coefficients from separate ALT-SR were used to assess the statin effect on the co-varying dynamic patterns of blood lipids and DNA methylation levels over time. Sex and baseline age were included as time-independent covariates.

The Bonferroni method was applied to control for multiple comparisons based on the numbers of independent tests. In the piecewise LGCM and ALT-SR model, three indices and their thresholds were used to indicate a well-fit model: chi-square value (*P* value > 0.05), root-mean-square error of approximation (RMSEA) (< 0.06), and comparative fit index (CFI) (> 0.95) [17]. Sensitivity analyses for all the aims were conducted by excluding samples with TG levels greater than 400 mg/dL to assess the impact of imputation of missing measurement of LDL.

All analyses were performed with R software (4.0.5). The Lavaan package (0.6–9) was used to fit the piecewise LGCM and ALT-SR model. Onyx software [18] was used to create the diagrams in Additional file 1: Figure S1 and Figure S2.

## Results

### Characteristics of the study sample

A total of 535 participants in SATSA with repeated measurements of DNA methylation and lipid levels were included in the analysis. The characteristics of the study sample are shown in Table 1. Among the 535 participants, 95 (18.0%) were statin users; of these, 43 (45.3%) started to use statins at IPT8, 20 (21.1%) started to use statins at IPT6, and 13.7% (13/95) and 10.5% (10/95) started to use statins at IPT5 and IPT9, respectively. Of note, three individuals were excluded from the analysis of the piecewise LGCM, as their data from the starting wave was missing. Correlations for MZ and DZ’s lipid and methylation levels are shown in Additional file 1: Table S3. Numbers of repeated measurements of blood lipids and DNA methylation at different IPTs are shown in Additional file 1: Table S4.

**Table 1:** Characteristics of the study population

Variables	N or Mean (median) value
N of individuals	535 (59 singletons, 83 MZ twin pairs, 155 DZ twin pairs)
Age (years), mean (SD)	68.2 (9.5)
Female (%)	313 (58.5)
Smoker (%)	94 (17.6)
TC (mmol/L), median (IQR)	6.1 (5.4–6.9)
LDL (mmol/L), median (IQR)	4.1 (3.6–4.8)
HDL (mmol/L), median (IQR)	1.4 (1.2–1.7)
TG (mmol/L), median (IQR)	1.3 (0.9–1.9)
Statin user (%)	95 (18.0)
Starting wave of statin use (%)	
IPT3	1 (1.1)
IPT5	13 (13.7)
IPT6	20 (21.1)
IPT8	43 (45.3)
IPT9	10 (10.5)
IPT10	5 (5.3)
Missing	3 (3.2)

Baseline values of age, smoker, TC, LDL, HDL and TG are shown. TC, LDL, HDL and TG are shown as median (interquartile range) because of their skewed distribution. MZ: monozygotic twins; DZ: dizygotic twins; IPT: in-person testing; TC: total cholesterol, LDL: low-density lipoprotein cholesterol; HDL: high-density lipoprotein cholesterol; TG: total triglyceride; SD: standard deviation; IQR: interquartile range

### Association between DNA methylation in candidate CpGs and blood lipids

We selected previously reported lipid-related CpGs as candidate CpGs (cg10177197, cg17901584 and cg27243685). In the mixed effect models (Table 2), cg17901584 annotated to *DHCR24* were positively associated with TC, LDL and HDL, whereas cg27243685 annotated to *ABCG1* were negatively associated with TC, LDL and HDL, and no significant associations were found for the two CpGs with TG. We did not find significant associations for cg10177197 with any of the lipids.

**Table 2:** Associations between blood lipids and DNA methylation from the mixed effect models

Dependent variables	Independent variables	Estimate	95% CI	*P* value
cg10177197	TC	− 0.001	− 0.003, 0.001	0.263
	LDL	− 0.003	− 0.005, 0.000	0.023
	HDL	− 0.007	− 0.014, 0.000	0.051
	TG	0.004	0.001, 0.006	0.015
cg17901584	TC	0.016	0.011, 0.020	4.70E−12
	LDL	0.014	0.009, 0.020	3.35E−07
	HDL	0.038	0.023, 0.054	2.46E−06
	TG	− 0.002	− 0.008, 0.005	0.631
cg27243685	TC	− 0.005	− 0.008, − 0.003	1.28E−06
	LDL	− 0.004	− 0.007, − 0.002	6.93E−04
	HDL	− 0.013	− 0.020, − 0.006	1.84E−04
	TG	0.003	0.000, 0.006	0.031

Age, sex and smoking were adjusted for as covariates. The Bonferroni adjusted significance level was set to 0.013 (0.05/4). TC: total cholesterol, LDL: low-density lipoprotein cholesterol; HDL: high-density lipoprotein cholesterol; TG: total triglyceride; CI: confidence interval

### Effect of statin therapy on the longitudinal trend of blood lipid levels

**Fig. 2:**
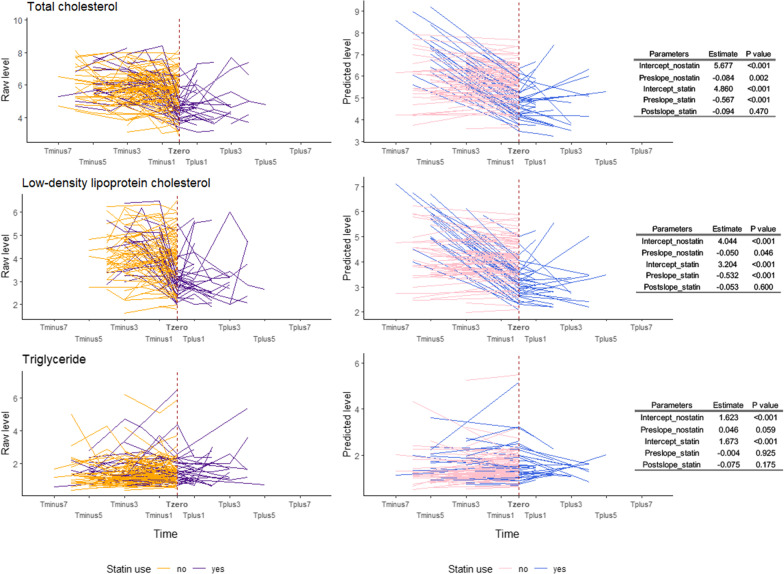
Longitudinal trajectory of serum lipids levels in response to statin treatment in Swedish male twins. This figure shows the longitudinal trajectory of serum lipids in response to statin therapy in Swedish male twins either in raw data or estimated from piecewise latent growth curve model (LGCM). The left part shows the longitudinal trend of raw lipid levels, where the yellow color represents the longitudinal trend of blood lipid for non-statin users and the purple line represents for statin users. The middle part is the longitudinal trend of predicted lipid levels derived from piecewise LGCM, similarly, the pink line represents the trend for non-statin users and the blue line is for statin users. Additional tables on the right list the estimates and *P* values of intercepts and slopes of predicted trajectories in the middle part for both statin users and non-users

**Fig. 3:**
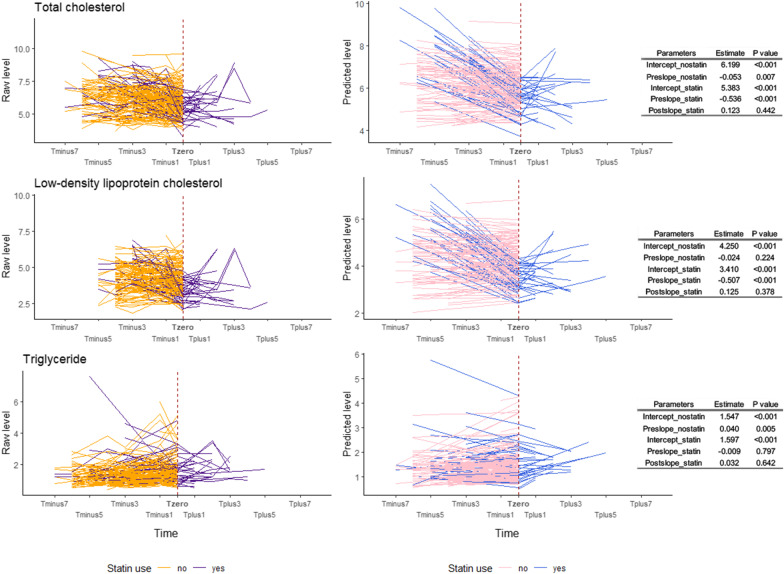
Longitudinal trajectory of serum lipids levels in response to statin treatment in Swedish female twins. This figure shows the longitudinal trajectory of serum lipids in response to statin therapy in Swedish female twins. The left three sub-figures show the longitudinal trend of raw lipid levels, where yellow lines represent the trend for non-statin users and the purple lines are for statin users. The middle sub-figures show the longitudinal trend of predicted lipid levels estimated from piecewise latent growth curve model (LGCM), where the purple lines represent the trend for non-statin users and the blue lines are for statin users. Tables on the right list the estimates of predicted trajectories (intercepts and slopes) and their *P* values for statin users and non-users

In the piecewise LGCM, we observed that TC and LDL in statin users significantly decreased from the first IPT until the time point when participants started to use statins (Pre_slope of TC for men: − 0.567, *P* < 0.001, for women: − 0.536, *P* < 0.001; Pre_slope of LDL for men: − 0.532, *P* < 0.001, for women: − 0.507, *P* < 0.001) and then did not change significantly in the phase after statin initiation (Post_slope of TC for men: − 0.094, *P* = 0.470, for women: 0.123, *P* = 0.442; Post_slope of LDL for men: − 0.053, *P* = 0.600, for women: 0.125, *P* = 0.378). TG did not show any significantly declining or rising trend either before or after statin therapy. For non-statin users, we found a statistically significant decreasing trend for TC both in men (Pre_slope: − 0.084, *P* = 0.002) and in women (Pre_slope: − 0.053, *P* = 0.007), while only in women for TG (Pre_slope: − 0.040, *P* = 0.005) (Figs. 2, 3).

The TC level at the knot (intercept) was significantly different between sexes (estimate: 0.522, *P* < 0.001) and between statin users and non-users (estimate: − 0.816, *P* < 0.001). The linear slope for TC before statin use differed significantly across different ages (estimate: − 0.007, *P* < 0.001) and was steeper in statin users compared to non-users (estimate: − 0.483, *P* < 0.001). The slope after statin initiation did not associate with age and sex. Similar patterns of associations between intercept, Pre_slope and Post_slope with covariates were found for LDL. We did not find any significant associations between intercept, slopes and covariates for TG. The piecewise model had the best fit for TG following the criteria of CFI and RMSEA. The piecewise model did not converge for HDL; therefore, the result was not shown (Table 3).

**Table 3:** Piecewise linear–linear growth curve models of latent intercept and slopes of longitudinal trends of blood lipid levels

Trait	Regressors	Intercept			Pre_slope			Post_slope			Model fit indices
		Estimate	95% CI	*P* value	Estimate	95% CI	*P* value	Estimate	95% CI	*P* value	
TC	Age	− 0.002	− 0.014, 0.010	0.741	− 0.007	− 0.011, − 0.004	< 0.001	− 0.023	− 0.049, 0.003	0.081	Chi-square value: 167.0 (DF: 43, * P* < 0.001), CFI: 0.812, RMSEA: 0.074
	Sex	0.522	0.316, 0.728	< 0.001	0.031	− 0.032, 0.094	0.342	0.217	− 0.183, 0.617	0.289	
	Statin	− 0.816	− 1.032, − 0.601	< 0.001	− 0.483	− 0.594, − 0.372	< 0.001	–	–	–	
LDL	Age	− 0.003	− 0.015, 0.009	0.589	− 0.005	− 0.008, − 0.001	0.007	− 0.020	− 0.045, 0.005	0.110	Chi-square value: 170.2 (DF: 55, < 0.001), CFI: 0.798, RMSEA: 0.063
	Sex	0.206	0.003, 0.409	0.047	0.025	− 0.036, 0.086	0.414	0.178	− 0.162, 0.518	0.305	
	Statin	− 0.840	− 1.048, − 0.632	< 0.001	− 0.483	− 0.598, − 0.367	< 0.001	–	–	–	
TG	Age	0.006	− 0.002, 0.014	0.114	0.001	− 0.002, 0.003	0.671	− 0.003	− 0.014, 0.007	0.550	Chi-square value: 97.2 (DF: 55, < 0.001), CFI: 0.914, RMSEA: 0.039
	Sex	− 0.077	− 0.253, 0.100	0.396	− 0.005	− 0.056, 0.045	0.832	0.107	− 0.032, 0.246	0.132	
	Statin	0.050	− 0.172, 0.272	0.658	− 0.050	− 0.117, 0.018	0.149	–	–	–	

Pre_slope means the slope before starting to use statins (for statin users) or the slope from the first observation to the last observation (nonstatin users); Post_slope means the slope after starting to use statins until the last observation (only for statin users). The Bonferroni adjusted significance level was set to 0.013 (0.05/4). TC: total cholesterol, LDL: low-density lipoprotein cholesterol; TG: total triglyceride; CI: confidence interval; DF: degree of freedom; CFI: comparative fit index; RMSEA: root-mean-square error of approximation

### Effect of statin therapy on the longitudinal trend of DNA methylation in candidate CpGs

**Fig. 4:**
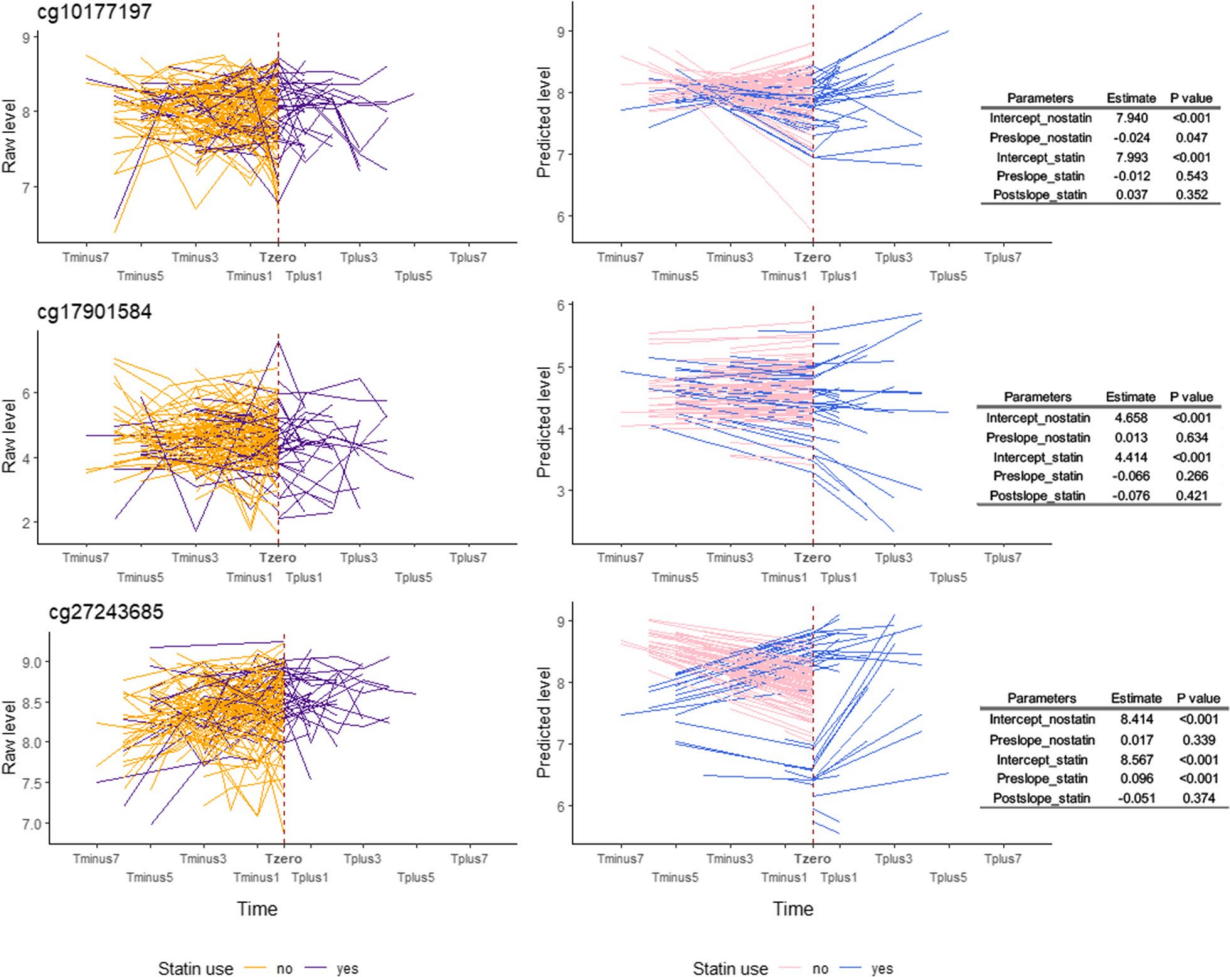
Longitudinal trajectory of DNA methylation levels in response to statin treatment in Swedish male twins. This figure shows the longitudinal trajectory of DNA methylation levels in response to statin therapy either in raw data (left part) or in predicted data estimated from piecewise latent growth curve model (middle part) in Swedish male twins. Yellow and purple lines shown in the left part of figure represent the longitudinal trend of raw DNA methylation levels for non-statin users and statin users, respectively. Similarly, pink and blue lines in the middle part represent the longitudinal trend of predicted DNA methylation levels derived from piecewise latent growth curve model for non-statin users and statin users. Tables on the right show estimates and *P* values of the intercepts and slopes of the predicted trajectories in statin users and non-users

**Fig. 5:**
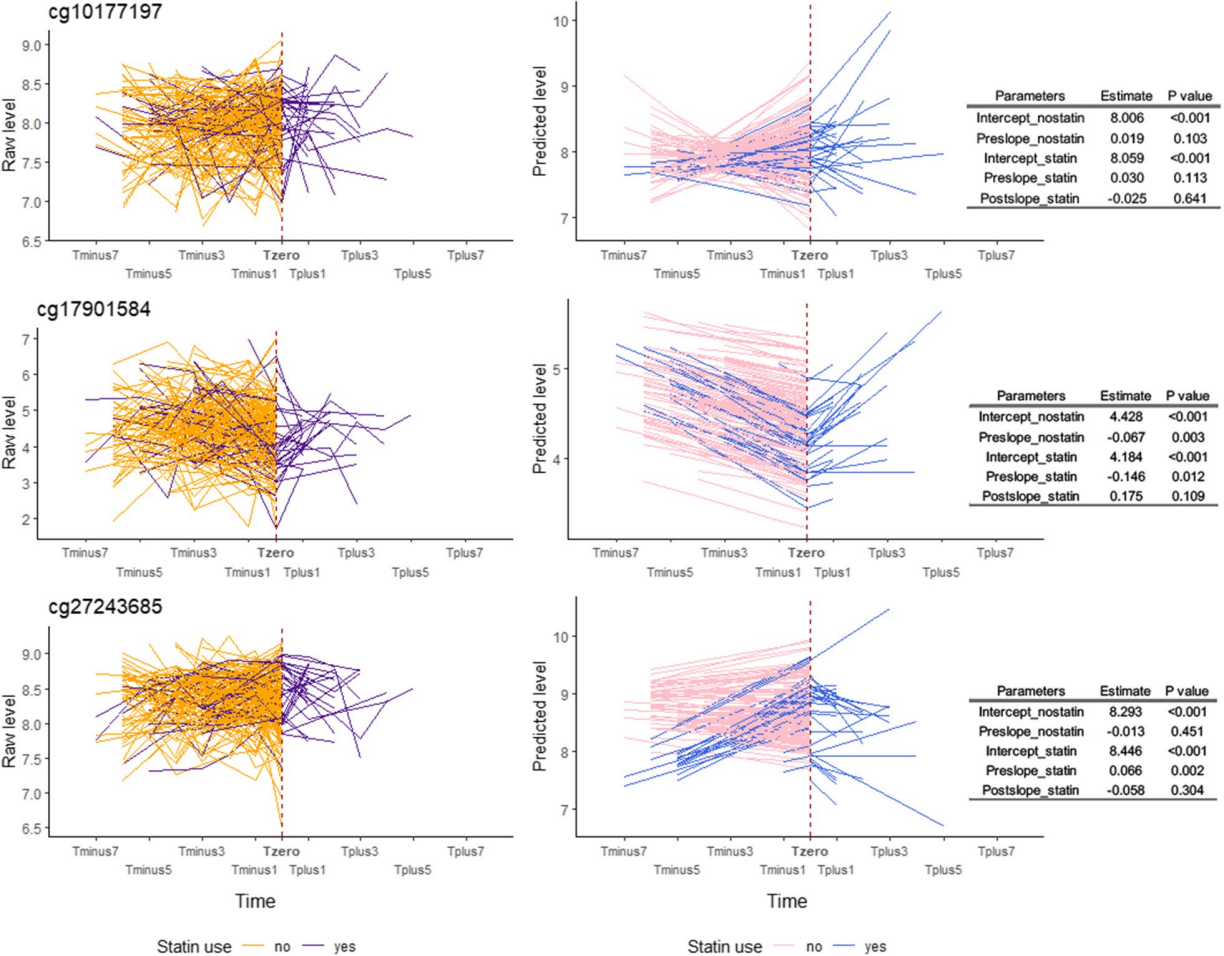
Longitudinal trajectory of DNA methylation levels in response to statin treatment in Swedish female twins. The figure shows the longitudinal trend of raw DNA methylation levels (left), the longitudinal trend of predicted DNA methylation levels derived from piecewise latent growth curve model (middle) for Swedish female twins. Yellow and purple lines represent the raw DNA methylation levels for non-statin users and statin users, respectively. Pink and blue lines represent the predicted DNA methylation levels for non-statin users and statin users, respectively. Tables show the estimates and *P* values of intercepts and slopes of the predicted trajectories (shown in middle sub-figures) in statin users and non-users

In statin users, we found a statistically significant increasing trend for DNA methylation level of cg27243685 before statin use (Pre_slope for men: 0.096, *P* < 0.001, and for women: 0.066, *P* = 0.002) and a decreasing trend for cg17901584 in women (Pre_slope: − 0.146, *P* = 0.012). No increasing or declining trend was found after statin initiation for the three CpGs. For non-statin users, we only observed significantly decreasing changes of DNA methylation levels over time for cg17901584 in female twins (Pre_slope: − 0.067, *P* = 0.003) (Figs. 4, 5).

DNA methylation level at the knot differed significantly between sexes for cg17901584 (estimate: − 0.230, *P* = 0.006) and cg27243685 (estimate: − 0.120, *P* = 0.001). Moreover, age (estimate: − 0.005, *P* = 0.009) and statin use (estimate: 0.153, *P* < 0.001) had different methylation level for cg27243685. The slope before statin use was associated with sex for cg10177197 (estimate: 0.042, *P* = 0.006) and cg17901584 (estimate: − 0.080, *P* = 0.012), while associated with statin use for cg27243685 (estimate: 0.079, *P* = 0.001). We did not find any significant associations between Post_slope and covariates for the three CpGs. The piecewise model had the best fit for cg17901584, and was only moderately good for the other CpGs (Table 4).

**Table 4:** Piecewise linear–linear growth curve models of latent intercept and slopes of longitudinal trends of DNA methylation on candidate CpGs

Trait	Regressors	Intercept			Pre_slope			Post_slope			Model fit indices
		Estimate	95% CI	*P* value	Estimate	95% CI	*P* value	Estimate	95% CI	*P* value	
cg10177197	Age	− 0.003	− 0.007, 0.001	0.214	− 0.001	− 0.003, 0.001	0.197	− 0.005	− 0.013, 0.003	0.258	Chi-square value: 45.9 (DF: 32, * P* = 0.053), CFI: 0.897, RMSEA: 0.029
	Sex	0.066	− 0.005, 0.138	0.070	0.042	0.012, 0.073	0.006	− 0.062	− 0.184, 0.059	0.316	
	Statin	0.053	− 0.030, 0.136	0.213	0.011	− 0.025, 0.048	0.539	–	–	–	
cg17901584	Age	0.008	0.000, 0.017	0.061	0.000	− 0.004, 0.003	0.807	− 0.005	− 0.021, 0.011	0.554	Chi-square value: 34.5 (DF: 32, * P* = 0.349), CFI: 0.957, RMSEA: 0.012
	Sex	− 0.230	− 0.393, − 0.067	0.006	− 0.080	− 0.142, − 0.017	0.012	0.251	0.003, 0.499	0.047	
	Statin	− 0.244	− 0.480, − 0.008	0.042	− 0.079	− 0.193, 0.036	0.177	–	–	–	
cg27243685	Age	− 0.005	− 0.009, − 0.001	0.009	0.000	− 0.003, 0.002	0.719	− 0.005	− 0.020, 0.011	0.561	Chi-square value: 38.1 (DF: 22, * P* = 0.018), CFI: 0.791, RMSEA: 0.037
	Sex	− 0.120	− 0.194, − 0.047	0.001	− 0.030	− 0.074, 0.015	0.190	− 0.007	− 0.151, 0.136	0.921	
	Statin	0.153	0.073, 0.232	< 0.001	0.079	0.031, 0.127	0.001	–	–	–	

Pre_slope means the slope in the phase from the first observation of not using statin until the observation of starting to use statin (for statin users) or the slope from the first observation to the last observation of repeated measurements (nonstatin users); Post_slope means the slope in the phase from the observation of starting to use statin until the last observation of repeated measurements (only for statin users). The Bonferroni adjusted significance level was set to 0.017 (0.05/3). CI: confidence interval; DF: degree of freedom; CFI: comparative fit index; RMSEA: root-mean-square error of approximation

### Effect of statin therapy on the co-varying pattern of blood lipid levels and DNA methylation in candidate CpGs

From the ALT-SR model using complete samples with men and women combined, we only observed that TG at IPT5 was significantly associated with DNA methylation level on cg27243685 at IPT6 in statin users (estimate = 0.383, *P* < 0.001). We did not find any other significant cross-lagged effects between lipid levels and methylation at CpGs. Detailed results from the cross-lagged effect model are shown in Additional file 1: Table S5.

### Sensitivity analyses

The models from sensitivity analyses of piecewise LGCM did not converge for cg10177197. The remaining results from sensitivity analyses were consistent with those from the primary analyses (Additional file 1: Tables S6-S9, Additional file 1: Figures S3-S6).

## Discussion

Our study has confirmed that statin treatment shows long-term efficacy in reducing lipid levels. Moreover, statin therapy is also associated with changes in DNA methylation levels at specific CpGs (for example, cg27243685 in the *ABCG1* gene), where the drug effect altered lipid levels which thereafter altered DNA methylation levels. We further analyzed the co-varying relationships between blood lipids, DNA methylation and statins using a longitudinal twin sample. Although results were mostly non-significant, we identified an association between TG levels and DNA methylation at a later follow-up in statin users. The findings from sensitivity analyses were consistent with those from the primary analyses and lead to similar conclusions about the statin effect. This study provides the first comprehensive assessment of the long-term patterns of change in lipid profiles and DNA methylation in response to statin therapy. It is important for a better understanding of the direction of effect, which changes come first, lipid or methylation, under the statin treatment. Moreover, it is important from a clinical perspective, for example, in what way DNA methylation could be used in future clinical practice.

In our analysis, we found significant associations between methylation level in cg17901584 and cg27243685 and blood lipids (TC, LDL and HDL), and some of the associations even reached suggestive genome-level significance [19]. Our results are to the most part in agreement with previous EWAS [2, 3, 20]. For example, the Rotterdam Study (n = 725 in the discovery population and n = 760 in the replication population) found that cg17901584 was positively associated with HDL and negatively associated with TG [2]. The other EWAS with larger sample size found several CpGs associated with lipid levels, which included cg17901584 and cg27243685 and have the same direction of the associations as we found [3, 20]. In our study, we also observed positive associations for DNA methylation levels in cg17901584 with HDL, and observed negative associations between cg27243685 and this lipid. cg17901584 is located at the TSS1500 (200–1500 bases upstream of transcription start site) of the *DHCR24* gene, and cg27243685 is located at the 5’UTR(untranslated region) or gene body of the *ABCG1* gene. The *DHCR24* gene encodes for the enzyme 3-hydroxysterol-24 reductase, which is involved in multiple pathways of producing cholesterol. The *ABCG1* gene encodes a protein which belongs to the superfamily of ATP-binding cassette (ABC) transporters and is involved in the reverse cholesterol transport pathway that efflux cholesterol to HDL. Therefore, associations between methylation in these genes and lipids are plausible. Other studies found that HDL was negatively associated with cg27243685, and this CpG was also negatively associated with *ABCG1* expression [3, 21]. Carolina Ochoa-Rosales et al. not only found a negative association between cg27243685 and *ABCG1* expression [5], but also observed a positive association between methylation of cg17901584 and *ABCG1* expression. Therefore, the different associations with *ABCG1* expression for the two CpGs may partly explain their opposite associations with lipids. Furthermore, from the perspective of biological mechanism, higher TG, LDL or TC levels and lower HDL might lead to the changes of DNA methylation that is associated with the expression of lipid associated genes and ultimately impact the lipid transport and metabolism. These findings suggest a role for epigenetics, that the cholesterol inhibits its own synthesis via the epigenetic pathway [22]. In terms of the complex feature of the epigenetic landscape, more studies are needed.

Moreover, we observed that statin users showed a steeper decline in TC and LDL levels compared to non-statin users. We also observed that statin therapy substantially decreased TC and LDL levels during the early phase after the initiation of statins, while the lipid levels remained stable during the follow-up time, which were similar to findings in previous studies [23–25]. The effect of statin use on the dynamic pattern of DNA methylation levels of the candidate CpGs was not as straightforward as the effect on blood lipids. For example, we only observed significant decreasing trends in the phase of pre-statin for cg17901584 in females and for cg27243685 in both female and male twins. Nonetheless, from estimates shown in Figs. 4–5 and comparing estimates to lipid trajectories in Figs. 2–3, we still observe that the absolute value of Post_slope for DNA methylation was overall larger than the value of Pre_slope, implying a “lagged” drug effect on the DNA methylation level of these CpGs.

To further scrutinize the directionality between blood lipids and methylation changes, the ALT-SR model was used to assess 84 cross-lagged effects of all lipid-CpG combinations (Additional file 1: Table S5). Then, in statin users we found TG levels to be associated with cg27243685 methylation levels in the following IPT after Bonferroni adjustment, indicating a possible temporal dynamics between blood lipids and DNA methylation. Additional cross-lagged effects were approaching borderline significance, for example, HDL at IPT8 and cg10177197 at IPT9 (*P* = 0.007) and cg27243685 at IPT6 and TG at IPT8 (*P* = 0.016), all in non-statin users. Previous studies have found inconsistent results regarding the direction in the causal pathway between lipids and DNA methylation. One EWAS study performed a stepwise Mendelian randomization analysis in 3,296 Dutch samples and found that blood lipids determined the methylation of genes related to lipid metabolism, and not vice versa [21]. In contrast, a bidirectional longitudinal association study with two years of follow-up time demonstrated significant cross-lagged effects between epigenetic age at an earlier time point and lipid levels at a later time point [26]. Our previous study conducted in the same cohort, using different CpGs and sub-samples, showed that the main type of cross-lagged effects go from DNA methylation of CVD related CpGs to lipids changes [16]. The inconsistency between studies may be caused by different reasons: different populations under study, sample sizes and selection of CpGs. For example, the CpGs used in this study were associated with blood lipids and statin use, which are different from the CpGs in our prior study selected for their association with CVD [16]. In addition, we used sub-samples (statin users and non-users) to do stratified ALT-SR analysis in this study, compared to the full sample used in the prior study. To summarize, the relationship between lipids and DNA methylation levels is complex. A recent large-scale genome-wide DNA methylation quantitative trait locus analysis may give some explanations [27]. The authors noted that it was difficult to determine the causal pathway between any specific CpG site and a trait. They concluded that it is likely due to common genetic variants influencing DNA methylation and traits independently, rather than by methylation being a mediator. In addition, the authors suggested that associations found in some previously reported EWAS were probably due to reverse causation, that the disease alters DNA methylation but not vice versa.

The key strength of this study is the rich sample characteristics and long duration. This study is based on a longitudinal twin cohort with nearly 30 years of follow-up time including repeated measurements of DNA methylation data. Hence, we could study the long-term covarying association between DNA methylation and blood lipids and explore the causal links therein. Twin studies represent a special type of epidemiological studies and they have certain assumptions that may challenge the validity of the results if they are not satisfied. Nonetheless, overall, results from twin cohorts can be generalizable to a wider population [28, 29]. The study also has several limitations. First, the sample size of the study was relatively small to fit the piecewise LGCM and ALT-SR model, especially for statin users, which is only 18% of the total sample. Moreover, the follow-up pattern of participants was diverse, which led to a large amount of missing data. Despite the limitations, some of the current results were still comparable to the results of previous studies. For example, we confirmed the findings from large EWAS for lipids [2, 3] (Table 2) and for statin use [5] (Additional file 1: Table S10). Second, we defined statin use in a simple way, i.e., non-statin/statin users. However, blood lipid levels are affected by adherence to statin use and different statin sub-types [30], and thus DNA methylation level may also be impacted. In the cohort, 12 participants changed their statin usage during the follow-up period and this might influence the results. Hence, we performed a sensitivity analysis by excluding those 12 participants. However, the piecewise LGCM models did not converge any more for TC, LDL, cg10177197 and cg27243685. The remaining results showed small differences from the results using the full data (results not shown). Finally, DNA methylation level is highly variant in a cell-specific or tissue-specific manner. We only have leukocyte DNA methylation from blood; therefore, the major findings based on genomic DNA methylation extracted from peripheral blood might be insufficient and future studies are needed.

## Conclusion

Our study sheds light on the potential effects of statin use on DNA methylation at three lipid-related candidate CpGs and on lipids, especially the longitudinal changing patterns. Further studies with larger sample sizes and with a multidisciplinary approach are warranted to highlight the epigenetic role in statin use.

## Supplementary Information

**Additional file 1.** **Figure S1**: Piecewise latent linear-linear growth curve model. This is a schematic diagram depicting the piecewise latent linear-linear growth curve model. The observed variables are shown as rectangles and latent variables as circles; the double-headed arrows represent variance or covariance of variables and single-headed arrows represent regression effects with the variable at the tail of the arrow having causal effect on the variable at the head. The regression effects are also called paths, directional effects and factor loadings, with the latter specifying the regression coefficients linking latent variables and observed variables. Tzero: the lipids (or DNA methylation) level at the time (denoted as in-person testing (IPT) in our study) of start to use statin. Tminus1-Tminus7: the lipids (or DNA methylation) level before statin treatment, and the suffix numbers of Tminus are determined by how many folds of the time interval deviating to Tzero. Tplus1-Tplus7: the lipids (or DNA methylation) level after statin treatment, and the suffix numbers have the same definition as that in Tminus. Intercept: the individual lipid (or DNA methylation) level at Tzero. Pre-slope: the changing rate of lipids (or DNA methylation) over time before the changing point for each individual. Post-slope: the changing rate of lipids (or DNA methylation) over time after the changing point for each individual. The factor loadings from the latent intercept to Tminus and Tplus variables are all set to 1. The factor loadings from latent “Pre_slope” are set as -7 to -1 for Tminus7 to Tminus1 and were equal to 0 for all Tplus variables. The factor loadings from the “Post_slope” to Tminus variables are all set as 0 but are set as 1 to 7 for Tplus1 to Tplus7, respectively. Baseline age, sex, and statin use are included as time-independent covariates as we assumed that these variables would have associations with latent intercept and slopes. **Figure S2**: Bivariate autoregressive latent trajectory model with structured residuals. This is a schematic diagram depicting the bivariate autoregressive latent trajectory model with structured residuals (ALT-SR). The observed variables are shown as rectangles (in purple) and latent variables as circles (in green); the double-headed arrows represent variance or covariance of variables and single-headed arrows represent regression effects with the variable at the tail of the arrow having causal effect on the variable at the head. The regression effects are also called paths, directional effects and factor loadings, with the latter specifying the regression coefficients linking latent variables and observed variables. The ALT-SR model is composed of the autoregressive model (AR) and the linear latent growth curve model (LGM). The LGM (the up and bottom parts) is established first by the latent intercept (mval.i and lipid.i in green circles) and the latent slope (mval.s and lipid.s in green circles) for DNA methylation and blood lipids, respectively. After that, AR (the middle part) is established by two paths: autoregressive path (red arrows) and cross-lagged path (blue arrows). mval.IPT3 to mval.IPT9: the observed variables measuring DNA methylation level at different time points for one specific CpG site. lipid.IPT3 to lipid.IPT9: the manifest variables measuring level of one specific blood lipids at different time points. mval.i and mval.s: latent intercept and slope for the DNA methylation trajectory. lipid.i and lipid.s: latent intercept and slope for the blood lipid trajectory. e.mval.IPT3 to e.mval.IPT9: latent residuals of DNA methylation at different time points. e.lipid.IPT3 to e.lipid.IPT9: latent residuals of blood lipid at different time points. The factor loadings from mval.i to mval.IPTs at different time points are all equal to 1, and the factor loadings from mval.s to these manifest variables are set as 0, 2, 3, 5 and 6 at IPT3, IPT5, IPT6, IPT8 and IPT9, respectively. The factor loadings for latent intercept and slope for blood lipids are set the same as that for DNA methylation. The factor loadings from residuals variables to manifest variables are all set as 1 (not shown in figure). Red arrows: autoregressive paths measuring the within-person changes of DNA methylation or blood lipids over time. Blue arrows: the cross-lagged paths measuring the predicting effect of DNA methylation at one time point on the within-person lipid level at the adjacent later time point, and/or vice versa. Dashed arrows: regression path from covariates to latent intercepts and slopes, and three variables (baseline age, sex, and statin use) are included as time-independent covariates. **Figure S3**: Sensitivity analysis of longitudinal trajectory of serum lipids levels in response to statin treatment in Swedish male twins by excluding samples with TG levels greater than 400 mg/dL. This figure shows the longitudinal trajectory of serum lipids in response to statin therapy in Swedish male twins either in raw data or estimated from piecewise latent growth curve model (LGCM) after excluding samples with TG levels greater than 400 mg/dL. The left part shows the longitudinal trend of raw lipid levels, where the yellow color represents the longitudinal trend of blood lipid for non-statin users and the purple line represents for statin users. The middle part is the longitudinal trend of predicted lipid levels derived from piecewise LGCM. Similarly, the pink line represents the trend for non-statin users and the blue line is for statin users. Additional tables on the right list the estimates and P values of intercepts and slopes of predicted trajectories in the middle part for both statin users and non-users. TG: total triglyceride. **Figure S4**: Sensitivity analysis of longitudinal trajectory of serum lipids levels in response to statin treatment in Swedish female twins by excluding samples with TG levels greater than 400 mg/dL. This figure shows the longitudinal trajectory of serum lipids in response to statin therapy in Swedish female twins either in raw data or estimated from piecewise latent growth curve model (LGCM) after excluding individuals with TG levels greater than 400 mg/dL. The left part shows the longitudinal trend of raw lipid levels, where the yellow color represents the longitudinal trend of blood lipid for non-statin users and the purple line represents for statin users. The middle part is the longitudinal trend of predicted lipid levels derived from piecewise LGCM, similarly, the pink line represents the trend for non-statin users and the blue line is for statin users. Additional tables on the right list the estimates and P values of intercepts and slopes of predicted trajectories in the middle part for both statin users and non-users. TG: total triglyceride. **Figure S5**: Sensitivity analysis of longitudinal trajectory of DNA methylation levels in response to statin treatment in Swedish male twins by excluding samples with TG levels greater than 400 mg/dL. The figure shows the longitudinal trend of raw DNA methylation levels (left), and the longitudinal trend of predicted DNA methylation levels derived from piecewise latent growth curve model (middle) for Swedish male twins after excluding samples with TG levels greater than 400 mg/dL. Yellow and purple lines represent the raw DNA methylation levels for non-statin users and statin users, respectively. Pink and blue lines represent the predicted DNA methylation levels for non-statin users and statin users, respectively. Tables show the estimates and P values of intercepts and slopes of the predicted trajectories (shown in middle sub-figures) in statin users and non-users. TG: total triglyceride. **Figure S6**: Sensitivity analysis of longitudinal trajectory of DNA methylation levels in response to statin treatment in Swedish female twins by excluding samples with TG levels greater than 400 mg/dL. The figure shows the longitudinal trend of raw DNA methylation levels (left), and the longitudinal trend of predicted DNA methylation levels derived from piecewise latent growth curve model (middle) for Swedish female twins after excluding samples with TG levels greater than 400 mg/dL. Yellow and purple lines represent the raw DNA methylation levels for non-statin users and statin users, respectively. Pink and blue lines represent the predicted DNA methylation levels for non-statin users and statin users, respectively. Tables show the estimates and P values of intercepts and slopes of the predicted trajectories (shown in middle sub-figures) in statin users and non-users. TG: total triglyceride.

## Data Availability

The datasets used in the current study are available in Array Express database of EMBL-EBL (www.ebi.ac.uk/arrayexpress, accession number E-MTAB-7309).
