# Supplementary material for: Dynamic patterns of blood lipids and DNA methylation in response to statin therapy
Source: Clin Epigenetics. 2022 Nov 28;14:153. doi: 10.1186/s13148-022-01375-8 (PMC9706978; doi:10.1186/s13148-022-01375-8)
Supplement: Supplementary file 1 — Additional file 1. Figure S1: Piecewise latent linear-linear growth curve model. This is a schematic diagram depicting the piecewise latent linear-linear growth curve model. The observed variables are shown as rectangles and latent variables as circles; the double-headed arrows represent variance or covariance of variables and single-headed arrows represent regression effects with the variable at the tail of the arrow having causal effect on the variable at the head. The regression effects are also called paths, directional effects and factor loadings, with the latter specifying the regression coefficients linking latent variables and observed variables. Tzero: the lipids (or DNA methylation) level at the time (denoted as in-person testing (IPT) in our study) of start to use statin. Tminus1-Tminus7: the lipids (or DNA methylation) level before statin treatment, and the suffix numbers of Tminus are determined by how many folds of the time interval deviating to Tzero. Tplus1-Tplus7: the lipids (or DNA methylation) level after statin treatment, and the suffix numbers have the same definition as that in Tminus. Intercept: the individual lipid (or DNA methylation) level at Tzero. Pre-slope: the changing rate of lipids (or DNA methylation) over time before the changing point for each individual. Post-slope: the changing rate of lipids (or DNA methylation) over time after the changing point for each individual. The factor loadings from the latent intercept to Tminus and Tplus variables are all set to 1. The factor loadings from latent “Pre_slope” are set as -7 to -1 for Tminus7 to Tminus1 and were equal to 0 for all Tplus variables. The factor loadings from the “Post_slope” to Tminus variables are all set as 0 but are set as 1 to 7 for Tplus1 to Tplus7, respectively. Baseline age, sex, and statin use are included as time-independent covariates as we assumed that these variables would have associations with latent intercept and slopes. Figure S2: Bivariate autoregressive la [file 13148_2022_1375_MOESM1_ESM.zip › supplementary file-revised version2/supple methods-revised version 2.docx]

**Supplementary Methods**

**Piecewise latent linear-linear growth curve model**

As an extension of latent growth curve model (LGCM) [1], piecewise LGCM breaks up the simple growth trend into separate linear segments that are connected together by knots (or change points or turning points). In this study, we chose the IPT of starting to use statins as the change point. Of note, there were two obvious phases of the change process separated by a change points for statin users; however, the piecewise latent model for non-statin users was reduced to one phase of the change process because there were no definable change points for these participants. For comparison purposes on the same time scale, we used the last measurement of IPT as the change point for nonstatin users. We constructed piecewise LGCM in the framework of a structural equation model (SEM). The depicted fitted model is presented in Supplementary Figure 1. As commonly shown in SEM diagrams, the observed variables are shown as rectangles and latent variables as circles; the double-headed arrows represent variance or covariance of variables and single-headed arrows represent regression effects with the variable at the tail of the arrow having causal effect on the variable at the head. The regression effects are also called paths, directional effects and factor loadings, with the latter specifying the regression coefficients linking latent variables and manifest variables.

To meet the assumption that the number of intervals and the time in-between must be the same for all individuals for LGCM model [1, 2], we first reorganized data on time metric for each participant, where “Tzero” represents the lipids (or methylation) levels at the IPT of start to use statins, and “Tminus” and “Tplus” represent the lipids (or methylation) levels before and after statin treatment, respectively. The suffix numbers of “Tminus” and “Tplus” are determined by number of intervals away from “Tzero” because the time interval between adjacent IPTs was approximately the same (2-3 years in SATSA). The details coding for “Tzero,” “Tplus,” and “Tminus” are presented in Supplementary Table 1 and Supplementary Table 2. In this study, the piecewise LGCM was established by three latent variables: one intercept (denoted as the Intercept in Supplementary Figure 1) measuring the individual lipid (or methylation) level at change points and two slopes (denoted as Pre_slope and Post_slope, respectively) measuring the change in lipids (or methylation) over time before and after the change point for each individual. The factor loadings from the latent intercept to “Tminus” and “Tplus” at different time points were set to 1. Factor loadings from latent “Pre_slope” were set as -7 to -1 for Tminus7 to Tminus1 and were equal to 0 for all “Tplus” variables. The factor loadings from the “Post_slope” to Tminus variables were all set as 0 but were set as 1 to 7 for Tplus1 to Tplus7, respectively. We also included sex (male=0, female=1), baseline age, and statin use (no=0, yes=1) as time-independent covariates in the regression equation of latent intercepts and slopes as we assumed that these covariates would have associations with these latent variables. Of note, baseline age was centered around its mean before entering into the model, and statin use as a covariate only has an association with Pre_slope but not with Post_slope because the Post_slope was only applicable in statin users. The genetic relatedness of twins within pairs was adjusted using the cluster option in the model. In the process of fitting the actual model, we found large proportions of missing data existing in some of the Tminus and Tplus variables, especially for those deviating further from Tzero (e.g., Tminus7, Tminus6, Tplus7, Tplus6); therefore, the model did not converge when using the full data. To solve this problem, we chose the maximum numbers of Tminus and Tplus variables with fewer missing data to fit the converged piecewise latent growth models for different lipids and CpGs. Moreover, we rescaled methylation data by multiplying the raw methylation data by 10 to ensure obtaining a converged model.

The Bonferroni method was applied to all analyses to control for multiple comparisons, and the adjusted significance level was set to 0.007 (0.05/7) on the basis of numbers of CpGs and types of blood lipids. We considered three fit indices and their thresholds to indicate a well-fit model: chi-square value (P value > 0.05), root-mean-square error of approximation (RMSEA) (<0.06), and comparative fit index (CFI) (>0.95) [3].

**Bivariate autoregressive latent trajectory model with structured residuals**

We fit the same bivariate autoregressive latent trajectory model with structured residuals (ALT-SR) model as in our previous research [4]. Details of the model are found here [5]. The ALT-SR model is also established in the framework of SEM, and the fitted model is displayed in Supplementary Figure 2. As shown, “mval” is the abbreviation for DNA methylation, therefore, mval.IPT3 to mval.IPT9 means the observed level of DNA methylation at different time points for one specific CpG site, and lipid.IPT3 to lipid.IPT9 represents the observed level of one specific blood lipids at different time points.

The ALT-SR model is composed of the autoregressive model (AR) and the linear latent growth curve model (LGM). The LGM was established first by the latent intercept and the latent slope which had the same meaning as in piecewise LGCM. The factor loading for latent intercept to manifest variables all equaled to 1 at different time points, and the factor loadings for latent slope were set as 0, 2, 3, 5 and 6 at IPT3, IPT5, IPT6, IPT8 and IPT9, respectively. IPT10 was not included because of more scarce methylation data at this time point which led to unconverged ALT-SR model. In AR, one regression path is called the autoregressive path (red arrows) which measures the within-person changes of DNA methylation or lipids over time, and the other is called a cross-lagged path (blue arrows), which measures the predicting effect of DNA methylation at one time point on the within-person lipid level at the adjacent subsequent time point, and/or vice versa. From the cross-lagged path, we can obtain information on the co-varying pattern of the blood lipids and DNA methylation across timelines. The set of time-independent covariates, solutions of the relatedness of twins and model inconvergence were the same as in piecewise LGCM. The Bonferroni adjusted significance level was set to 0.004 (0.05/12) based on the numbers of different combinations of CpGs and blood lipids.

References

1. Kohli, N. and J.R. Harring, *Modeling Growth in Latent Variables Using a Piecewise Function.* Multivariate Behavioral Research, 2013. **48**(3): p. 370-397.

2. Byrne, B.M. and G. Crombie, *Modeling and Testing Change: An Introduction to the Latent Growth Curve Model.* Understanding Statistics, 2003. **2**(3): p. 177-203.

3. Hu, L.t. and P.M. Bentler, *Cutoff criteria for fit indexes in covariance structure analysis: Conventional criteria versus new alternatives.* Structural Equation Modeling: A Multidisciplinary Journal, 1999. **6**(1): p. 1-55.

4. Qin, X., et al., *The epigenetic etiology of cardiovascular disease in a longitudinal Swedish twin study.* Clinical Epigenetics, 2021. **13**(1): p. 129.

5. Curran, P.J., et al., *The separation of between-person and within-person components of individual change over time: a latent curve model with structured residuals.* J Consult Clin Psychol, 2014. **82**(5): p. 879-94.
