# Supplementary material for: Dynamic patterns of blood lipids and DNA methylation in response to statin therapy
Source: Clin Epigenetics. 2022 Nov 28;14:153. doi: 10.1186/s13148-022-01375-8 (PMC9706978; doi:10.1186/s13148-022-01375-8)
Supplement: Supplementary file 1 — Additional file 1. Figure S1: Piecewise latent linear-linear growth curve model. This is a schematic diagram depicting the piecewise latent linear-linear growth curve model. The observed variables are shown as rectangles and latent variables as circles; the double-headed arrows represent variance or covariance of variables and single-headed arrows represent regression effects with the variable at the tail of the arrow having causal effect on the variable at the head. The regression effects are also called paths, directional effects and factor loadings, with the latter specifying the regression coefficients linking latent variables and observed variables. Tzero: the lipids (or DNA methylation) level at the time (denoted as in-person testing (IPT) in our study) of start to use statin. Tminus1-Tminus7: the lipids (or DNA methylation) level before statin treatment, and the suffix numbers of Tminus are determined by how many folds of the time interval deviating to Tzero. Tplus1-Tplus7: the lipids (or DNA methylation) level after statin treatment, and the suffix numbers have the same definition as that in Tminus. Intercept: the individual lipid (or DNA methylation) level at Tzero. Pre-slope: the changing rate of lipids (or DNA methylation) over time before the changing point for each individual. Post-slope: the changing rate of lipids (or DNA methylation) over time after the changing point for each individual. The factor loadings from the latent intercept to Tminus and Tplus variables are all set to 1. The factor loadings from latent “Pre_slope” are set as -7 to -1 for Tminus7 to Tminus1 and were equal to 0 for all Tplus variables. The factor loadings from the “Post_slope” to Tminus variables are all set as 0 but are set as 1 to 7 for Tplus1 to Tplus7, respectively. Baseline age, sex, and statin use are included as time-independent covariates as we assumed that these variables would have associations with latent intercept and slopes. Figure S2: Bivariate autoregressive la [file 13148_2022_1375_MOESM1_ESM.zip › supplementary file-revised version2/supplementary tables-revised version 2.docx]

Supplementary table 1: Coding rule to reorganize the repeated measurement of lipids and methylation levels according to when to start the statin therapy in **statin users** in piecewise linear-linear growth curve model

| IPT when statin therapy started | ***Tzero*** | **Tminus1** | **Tminus2** | **Tminus3** | **Tminus4** | **Tminus5** | **Tminus6** | **Tminus7** | **Tplus1** | **Tplus2** | **Tplus3** | **Tplus4** | **Tplus5** | **Tplus6** | **Tplus7** |
| --- | --- | --- | --- | --- | --- | --- | --- | --- | --- | --- | --- | --- | --- | --- | --- |
| IPT3 | ***IPT3*** | NA | NA | NA | NA | NA | NA | NA | NA | IPT5 | IPT6 | NA | IPT8 | IPT9 | IPT10 |
| IPT5 | ***IPT5*** | NA | IPT3 | NA | NA | NA | NA | NA | IPT6 | NA | IPT8 | IPT9 | IPT10 | NA | NA |
| IPT6 | ***IPT6*** | IPT5 | NA | IPT3 | NA | NA | NA | NA | NA | IPT8 | IPT9 | IPT10 | NA | NA | NA |
| IPT8 | ***IPT8*** | NA | IPT6 | IPT5 | NA | IPT3 | NA | NA | IPT9 | IPT10 | NA | NA | NA | NA | NA |
| IPT9 | ***IPT9*** | IPT8 | NA | IPT6 | IPT5 | NA | IPT3 | NA | IPT10 | NA | NA | NA | NA | NA | NA |
| IPT10 | ***IPT10*** | IPT9 | IPT8 | NA | IPT6 | IPT5 | NA | IPT3 | NA | NA | NA | NA | NA | NA | NA |

Note: To fit a piecewise latent growth model for statin users, the changing time point was defined as IPT when statin therapy started. Tzero was defined as lipids (methylation levels) at IPT when to start to use statin; Tminus1-Tminus7 mean the lipids (or methylation) levels before statin treatment, and the suffix number after “Tminus” was determined by how many folds of the time interval deviating to “Tzero”; Tplus1-Tplus7 mean the lipids (or methylation) levels after statin treatment and it followed the same coding rule for the suffix number. IPT: in-person testing.

Supplementary table 2: Coding rule to reorganize the repeated measurement of lipids and methylation levels according to when start the statin therapy in **non-statin users** in piecewise linear-linear growth curve model

| Last IPT | ***Tzero*** | **Tminus1** | **Tminus2** | **Tminus3** | **Tminus4** | **Tminus5** | **Tminus6** | **Tminus7** |
| --- | --- | --- | --- | --- | --- | --- | --- | --- |
| IPT3 | ***IPT3*** | NA | NA | NA | NA | NA | NA | NA |
| IPT5 | ***IPT5*** | NA | IPT3 | NA | NA | NA | NA | NA |
| IPT6 | ***IPT6*** | IPT5 | NA | IPT3 | NA | NA | NA | NA |
| IPT8 | ***IPT8*** | NA | IPT6 | IPT5 | NA | IPT3 | NA | NA |
| IPT9 | ***IPT9*** | IPT8 | NA | IPT6 | IPT5 | NA | IPT3 | NA |
| IPT10 | ***IPT10*** | IPT9 | IPT8 | NA | IPT6 | IPT5 | NA | IPT3 |

Note: Since there was no definable changing points for non-statin users, the changing time point was defined as the last IPT of repeated measurements. Tzero was defined as lipids (methylation levels) at last observed IPT; Tminus1-Tminus7 mean the lipids (or methylation) levels after statin treatment, and the suffix number after “Tminus” was determined by how many folds of the time interval deviating to “Tzero”. IPT: in-person testing.

Supplementary table 3: Twin correlations of lipids and DNA methylation

|  | Intraclass correlation | |
| --- | --- | --- |
|  | MZ | DZ |
| TC | 0.660 | 0.300 |
| LDL | 0.597 | 0.149 |
| HDL | 0.703 | 0.381 |
| TG | 0.450 | 0.211 |
| cg10177197 | 0.012 | 0.153 |
| cg17901584 | 0.259 | 0.219 |
| cg27243685 | 0.189 | 0.236 |

Note: TC: total cholesterol, LDL: low-density lipoprotein cholesterol; HDL: high-density lipoprotein cholesterol; TG: total triglyceride, MZ: monozygotic twins; DZ: dizygotic twins.

Supplementary table 4: Numbers of measurements of lipids and DNA methylation at different IPTs

| IPTs | Statin use | TC | TG | LDL | HDL | DNA methylation (3 CpGs) |
| --- | --- | --- | --- | --- | --- | --- |
| IPT3 | Yes | 43 | 43 | 0 | 0 | 44 |
|  | No | 264 | 264 | 0 | 0 | 275 |
| IPT5 | Yes | 60 | 60 | 60 | 60 | 61 |
|  | No | 271 | 271 | 271 | 271 | 272 |
| IPT6 | Yes | 55 | 55 | 55 | 55 | 59 |
|  | No | 172 | 172 | 172 | 172 | 186 |
| IPT8 | Yes | 69 | 68 | 69 | 69 | 71 |
|  | No | 174 | 174 | 174 | 174 | 174 |
| IPT9 | Yes | 62 | 62 | 62 | 62 | 62 |
|  | No | 135 | 135 | 135 | 135 | 137 |
| IPT10 | Yes | 23 | 23 | 23 | 23 | 23 |
|  | No | 19 | 19 | 19 | 19 | 19 |

Note: TC and TG, LDL and HDL, DNA methylation of three CpGs have the same numbers of repeated measurements according to IPTs, respectively. IPT: in-person testing; TC: total cholesterol, LDL: low-density lipoprotein cholesterol; HDL: high-density lipoprotein cholesterol; TG: total triglyceride

Supplementary table 5: Cross-lagged effect between blood lipids and DNA methylation on candidate CpGs over adjacent IPTs from bivariate autoregressive latent trajectory model with structured residuals

| Dependent variable | Independent variable | Statin users | | |  | Non-statin users | | |
| --- | --- | --- | --- | --- | --- | --- | --- | --- |
|  |  | Estimate | 95% CI | P value |  | Estimate | 95% CI | P value |
| cg10177197.IPT5 | TC.IPT3 | -0.032 | -0.193, 0.129 | 0.696 |  | 0.012 | -0.113, 0.138 | 0.848 |
| cg10177197.IPT6 | TC.IPT5 | -0.107 | -0.264, 0.050 | 0.181 |  | 0.119 | -0.072, 0.311 | 0.222 |
| cg10177197.IPT8 | TC.IPT6 | 0.002 | -0.184, 0.187 | 0.986 |  | -0.008 | -0.43, 0.413 | 0.969 |
| cg10177197.IPT9 | TC.IPT8 | -0.177 | -0.441, 0.086 | 0.188 |  | 0.033 | -0.165, 0.231 | 0.745 |
| TC.IPT5 | cg10177197.IPT3 | -0.038 | -0.783, 0.706 | 0.919 |  | -0.158 | -1.062, 0.745 | 0.731 |
| TC.IPT6 | cg10177197.IPT5 | -1.457 | -2.659, -0.255 | 0.017 |  | -0.165 | -0.488, 0.158 | 0.317 |
| TC.IPT8 | cg10177197.IPT6 | 0.347 | -0.189, 0.884 | 0.204 |  | 0.166 | -0.464, 0.795 | 0.606 |
| TC.IPT9 | cg10177197.IPT8 | -0.356 | -1.121, 0.409 | 0.362 |  | -0.253 | -0.587, 0.082 | 0.138 |
| cg17901584.IPT5 | TC.IPT3 | -0.171 | -0.661, 0.319 | 0.494 |  | 0.025 | -0.226, 0.277 | 0.843 |
| cg17901584.IPT6 | TC.IPT5 | -0.118 | -0.596, 0.360 | 0.630 |  | 0.091 | -0.36, 0.542 | 0.692 |
| cg17901584.IPT8 | TC.IPT6 | -0.041 | -0.381, 0.299 | 0.812 |  | 0.078 | -0.408, 0.565 | 0.752 |
| cg17901584.IPT9 | TC.IPT8 | -0.105 | -0.422, 0.211 | 0.514 |  | 0.356 | -0.097, 0.810 | 0.123 |
| TC.IPT5 | cg17901584.IPT3 | 0.123 | -0.093, 0.339 | 0.264 |  | 0.325 | 0.015, 0.634 | 0.040 |
| TC.IPT6 | cg17901584.IPT5 | -0.077 | -0.415, 0.262 | 0.658 |  | 0.186 | -0.004, 0.375 | 0.055 |
| TC.IPT8 | cg17901584.IPT6 | 0.041 | -0.532, 0.614 | 0.889 |  | -0.244 | -0.524, 0.037 | 0.089 |
| TC.IPT9 | cg17901584.IPT8 | -0.051 | -0.355, 0.253 | 0.741 |  | -0.069 | -0.24, 0.103 | 0.433 |
| cg27243685.IPT5 | TC.IPT3 | -0.017 | -0.162, 0.127 | 0.813 |  | -0.096 | -0.245, 0.054 | 0.212 |
| cg27243685.IPT6 | TC.IPT5 | 0.052 | -0.197, 0.301 | 0.683 |  | 0.056 | -0.136, 0.248 | 0.569 |
| cg27243685.IPT8 | TC.IPT6 | 0.009 | -0.111, 0.130 | 0.879 |  | 0.011 | -0.154, 0.176 | 0.898 |
| cg27243685.IPT9 | TC.IPT8 | 0.094 | -0.070, 0.257 | 0.263 |  | -0.175 | -0.437, 0.086 | 0.189 |
| TC.IPT5 | cg27243685.IPT3 | -0.477 | -1.421, 0.467 | 0.322 |  | 0.238 | -0.320, 0.796 | 0.403 |
| TC.IPT6 | cg27243685.IPT5 | -0.444 | -1.916, 1.029 | 0.555 |  | 0.063 | -0.266, 0.392 | 0.709 |
| TC.IPT8 | cg27243685.IPT6 | -0.118 | -1.395, 1.159 | 0.856 |  | 0.273 | -0.412, 0.958 | 0.435 |
| TC.IPT9 | cg27243685.IPT8 | 0.585 | -1.211, 2.381 | 0.523 |  | -0.132 | -0.440, 0.176 | 0.402 |
| cg10177197.IPT6 | LDL.IPT5 | -0.027 | -0.237, 0.184 | 0.802 |  | 0.226 | -0.324, 0.777 | 0.421 |
| cg10177197.IPT8 | LDL.IPT6 | -0.036 | -0.244, 0.171 | 0.732 |  | 0.041 | -0.329, 0.411 | 0.829 |
| cg10177197.IPT9 | LDL.IPT8 | -0.171 | -0.459, 0.118 | 0.246 |  | 0.015 | -0.450, 0.480 | 0.949 |
| LDL.IPT6 | cg10177197.IPT5 | -0.994 | -3.677, 1.688 | 0.468 |  | 0.177 | -0.672, 1.026 | 0.682 |
| LDL.IPT8 | cg10177197.IPT6 | 0.629 | -0.508, 1.767 | 0.278 |  | 0.102 | -0.892, 1.096 | 0.840 |
| LDL.IPT9 | cg10177197.IPT8 | -0.370 | -1.514, 0.775 | 0.527 |  | -0.079 | -0.607, 0.45 | 0.771 |
| cg17901584.IPT6 | LDL.IPT5 | -0.142 | -0.706, 0.421 | 0.621 |  | 0.329 | -0.505, 1.163 | 0.439 |
| cg17901584.IPT8 | LDL.IPT6 | 0.008 | -0.542, 0.557 | 0.978 |  | 0.089 | -0.621, 0.799 | 0.806 |
| cg17901584.IPT9 | LDL.IPT8 | -0.190 | -0.482, 0.101 | 0.200 |  | 0.328 | -0.175, 0.831 | 0.202 |
| LDL.IPT6 | cg17901584.IPT5 | -0.320 | -1.200, 0.559 | 0.476 |  | 0.246 | -0.128, 0.619 | 0.198 |
| LDL.IPT8 | cg17901584.IPT6 | 0.013 | -0.509, 0.534 | 0.962 |  | -0.233 | -0.514, 0.049 | 0.105 |
| LDL.IPT9 | cg17901584.IPT8 | -0.219 | -0.673, 0.235 | 0.344 |  | 0.012 | -0.239, 0.264 | 0.923 |
| cg27243685.IPT6 | LDL.IPT5 | 0.016 | -0.277, 0.310 | 0.913 |  | -0.105 | -0.439, 0.230 | 0.539 |
| cg27243685.IPT8 | LDL.IPT6 | 0.022 | -0.106, 0.151 | 0.734 |  | -0.049 | -0.31, 0.213 | 0.714 |
| cg27243685.IPT9 | LDL.IPT8 | 0.150 | -0.030, 0.331 | 0.103 |  | -0.252 | -0.505, 0.001 | 0.051 |
| LDL.IPT6 | cg27243685.IPT5 | -0.302 | -1.695, 1.090 | 0.670 |  | -0.145 | -0.893, 0.602 | 0.704 |
| LDL.IPT8 | cg27243685.IPT6 | 0.232 | -1.757, 2.222 | 0.819 |  | 0.001 | -0.621, 0.624 | 0.997 |
| LDL.IPT9 | cg27243685.IPT8 | 0.807 | -0.368, 1.983 | 0.178 |  | -0.541 | -1.097, 0.016 | 0.057 |
| cg10177197.IPT6 | HDL.IPT5 | -0.987 | -2.644, 0.671 | 0.243 |  | 3.729 | -8.105, 15.564 | 0.537 |
| cg10177197.IPT8 | HDL.IPT6 | -0.249 | -1.620, 1.122 | 0.722 |  | 0.804 | 0.028, 1.58 | 0.042 |
| cg10177197.IPT9 | HDL.IPT8 | 0.108 | -1.467, 1.684 | 0.893 |  | 1.033 | 0.289, 1.778 | 0.007 |
| HDL.IPT6 | cg10177197.IPT5 | -0.229 | -0.653, 0.194 | 0.289 |  | 0.323 | -0.919, 1.565 | 0.610 |
| HDL.IPT8 | cg10177197.IPT6 | -0.176 | -0.397, 0.046 | 0.119 |  | 0.250 | -0.089, 0.589 | 0.148 |
| HDL.IPT9 | cg10177197.IPT8 | -0.039 | -0.402, 0.325 | 0.836 |  | 0.226 | -0.037, 0.489 | 0.092 |
| cg17901584.IPT6 | HDL.IPT5 | - | - | - |  | 5.832 | -19.975, 31.638 | 0.658 |
| cg17901584.IPT8 | HDL.IPT6 | - | - | - |  | 0.348 | -0.883, 1.579 | 0.579 |
| cg17901584.IPT9 | HDL.IPT8 | - | - | - |  | 0.739 | -1.595, 3.072 | 0.535 |
| HDL.IPT6 | cg17901584.IPT5 | - | - | - |  | 0.804 | -4.174, 5.782 | 0.752 |
| HDL.IPT8 | cg17901584.IPT6 | - | - | - |  | -0.029 | -0.074, 0.015 | 0.195 |
| HDL.IPT9 | cg17901584.IPT8 | - | - | - |  | 0.061 | -0.057, 0.179 | 0.310 |
| cg27243685.IPT6 | HDL.IPT5 | - | - | - |  | -9.105 | -40.531, 22.32 | 0.570 |
| cg27243685.IPT8 | HDL.IPT6 | - | - | - |  | -0.116 | -1.053, 0.820 | 0.808 |
| cg27243685.IPT9 | HDL.IPT8 | - | - | - |  | -0.764 | -2.499, 0.972 | 0.388 |
| HDL.IPT6 | cg27243685.IPT5 | - | - | - |  | -0.886 | -2.461, 0.689 | 0.270 |
| HDL.IPT8 | cg27243685.IPT6 | - | - | - |  | -0.044 | -0.248, 0.161 | 0.676 |
| HDL.IPT9 | cg27243685.IPT8 | - | - | - |  | -0.234 | -0.560, 0.091 | 0.158 |
| cg10177197.IPT5 | TG.IPT3 | 0.438 | -2.817, 3.693 | 0.792 |  | -0.199 | -0.91, 0.512 | 0.583 |
| cg10177197.IPT6 | TG.IPT5 | 0.223 | -0.371, 0.816 | 0.462 |  | 0.034 | -0.366, 0.435 | 0.866 |
| cg10177197.IPT8 | TG.IPT6 | 0.010 | -0.246, 0.265 | 0.941 |  | 0.103 | -0.323, 0.528 | 0.637 |
| cg10177197.IPT9 | TG.IPT8 | -0.053 | -0.408, 0.302 | 0.770 |  | 0.004 | -0.442, 0.449 | 0.986 |
| TG.IPT5 | cg10177197.IPT3 | 0.735 | -3.138, 4.608 | 0.710 |  | -0.437 | -2.593, 1.720 | 0.691 |
| TG.IPT6 | cg10177197.IPT5 | 0.044 | -1.864, 1.953 | 0.964 |  | 0.167 | -0.158, 0.492 | 0.313 |
| TG.IPT8 | cg10177197.IPT6 | -0.006 | -1.81, 1.799 | 0.995 |  | -0.307 | -1.61, 0.996 | 0.644 |
| TG.IPT9 | cg10177197.IPT8 | -0.225 | -0.844, 0.395 | 0.478 |  | -0.285 | -0.617, 0.046 | 0.092 |
| cg17901584.IPT5 | TG.IPT3 | -0.507 | -1.896, 0.882 | 0.475 |  | -0.107 | -0.387, 0.173 | 0.456 |
| cg17901584.IPT6 | TG.IPT5 | -0.310 | -1.163, 0.543 | 0.477 |  | 0.166 | -0.115, 0.446 | 0.247 |
| cg17901584.IPT8 | TG.IPT6 | -0.563 | -1.298, 0.173 | 0.134 |  | 0.189 | -0.559, 0.937 | 0.620 |
| cg17901584.IPT9 | TG.IPT8 | 0.104 | -0.267, 0.476 | 0.582 |  | 0.254 | -0.152, 0.660 | 0.220 |
| TG.IPT5 | cg17901584.IPT3 | -0.373 | -0.817, 0.071 | 0.099 |  | -0.012 | -0.295, 0.271 | 0.934 |
| TG.IPT6 | cg17901584.IPT5 | 0.127 | -0.118, 0.373 | 0.309 |  | -0.037 | -0.208, 0.133 | 0.669 |
| TG.IPT8 | cg17901584.IPT6 | -0.320 | -1.093, 0.453 | 0.416 |  | -0.048 | -0.317, 0.222 | 0.729 |
| TG.IPT9 | cg17901584.IPT8 | -0.082 | -0.286, 0.123 | 0.433 |  | -0.088 | -0.207, 0.032 | 0.150 |
| cg27243685.IPT5 | TG.IPT3 | 0.049 | -0.115, 0.213 | 0.556 |  | -0.079 | -0.280, 0.121 | 0.438 |
| **cg27243685.IPT6** | **TG.IPT5** | **0.383** | **0.173, 0.594** | **<0.001** |  | -0.154 | -0.321, 0.013 | 0.072 |
| cg27243685.IPT8 | TG.IPT6 | -0.153 | -0.501, 0.196 | 0.391 |  | -0.137 | -0.391, 0.118 | 0.294 |
| cg27243685.IPT9 | TG.IPT8 | -0.030 | -0.275, 0.215 | 0.809 |  | -0.087 | -0.385, 0.21 | 0.565 |
| TG.IPT5 | cg27243685.IPT3 | 0.582 | 0.019, 1.146 | 0.043 |  | -0.197 | -0.531, 0.137 | 0.248 |
| TG.IPT6 | cg27243685.IPT5 | -0.349 | -1.082, 0.385 | 0.352 |  | -0.081 | -0.578, 0.416 | 0.750 |
| TG.IPT8 | cg27243685.IPT6 | 1.174 | -0.575, 2.923 | 0.188 |  | -0.498 | -0.904, -0.091 | 0.016 |
| TG.IPT9 | cg27243685.IPT8 | -0.230 | -1.234, 0.775 | 0.654 |  | -0.073 | -0.366, 0.220 | 0.625 |

Baseline age, sex and smoking were adjusted as covariates. TC: total cholesterol, LDL: low-density lipoprotein cholesterol; HDL: high-density lipoprotein cholesterol; TG: total triglyceride; CI: confidence interval; IPT: in-person testing. “-” means we did not get converged result from this bivariate autoregressive latent trajectory model with structured residuals.

Supplementary table 6: Sensitivity analysis of associations between blood lipids and DNA methylation from the mixed effect models by excluding samples with TG levels great than 400 mg/dL.

| Dependent variables | Independent variables | Estimate | 95% CI | P value |
| --- | --- | --- | --- | --- |
| cg10177197 | TC | -0.001 | -0.003, 0.001 | 0.262 |
|  | LDL | -0.003 | -0.005, 0.000 | 0.040 |
|  | HDL | -0.007 | -0.014, 0.000 | 0.043 |
|  | TG | 0.005 | 0.001, 0.008 | 0.006 |
| cg17901584 | TC | 0.016 | 0.011, 0.020 | 2.37E-11 |
|  | LDL | 0.014 | 0.008, 0.019 | 2.03E-06 |
|  | HDL | 0.038 | 0.022, 0.054 | 3.98E-06 |
|  | TG | -0.003 | -0.011, 0.004 | 0.432 |
| cg27243685 | TC | -0.006 | -0.008, -0.003 | 7.63E-07 |
|  | LDL | -0.004 | -0.007, -0.002 | 6.60E-04 |
|  | HDL | -0.014 | -0.021, -0.006 | 1.90E-04 |
|  | TG | 0.004 | 0.001, 0.008 | 0.020 |

Age, sex and smoking were adjusted for as covariates. The Bonferroni adjusted significance level was set to 0.013 (0.05/4). TC: total cholesterol, LDL: low-density lipoprotein cholesterol; HDL: high-density lipoprotein cholesterol; TG: total triglyceride; CI: confidence interval.

Supplementary table 7: Sensitivity analysis of piecewise linear-linear growth curve models of latent intercept and slopes of longitudinal trends of blood lipid levels by excluding samples with TG levels great than 400 mg/dL.

| Trait | Regressors | Intercept | | |  | Pre_slope | | |  | Post_slope | | | Model fit indices |
| --- | --- | --- | --- | --- | --- | --- | --- | --- | --- | --- | --- | --- | --- |
|  |  | Estimate | 95% CI | P value |  | Estimate | 95% CI | P value |  | Estimate | 95% CI | P value |  |
| TC | Age | -0.002 | -0.013, 0.010 | 0.736 |  | -0.007 | -0.011, -0.004 | <0.001 |  | -0.025 | -0.050, 0.001 | 0.055 | Chi-square value: 167.1 (DF: 43, P<0.001), CFI: 0.806, RMSEA: 0.075 |
|  | Sex | 0.522 | 0.314, 0.729 | <0.001 |  | 0.032 | -0.032, 0.096 | 0.329 |  | 0.263 | -0.155, 0.681 | 0.218 |  |
|  | Statin | -0.809 | -1.026, -0.592 | <0.001 |  | -0.492 | -0.607, -0.377 | <0.001 |  | -- | -- | -- |  |
| LDL | Age | -0.004 | -0.015, 0.008 | 0.550 |  | -0.005 | -0.008, -0.001 | 0.009 |  | -0.020 | -0.043, 0.004 | 0.101 | Chi-square value: 171.9 (DF: 55, <0.001), CFI: 0.789, RMSEA: 0.065 |
|  | Sex | 0.197 | -0.005, 0.399 | 0.056 |  | 0.023 | -0.038, 0.085 | 0.458 |  | 0.156 | -0.199, 0.511 | 0.389 |  |
|  | Statin | -0.836 | -1.047, -0.624 | <0.001 |  | -0.497 | -0.618, -0.375 | <0.001 |  | -- | -- | -- |  |
| TG | Age | 0.008 | 0.001, 0.015 | 0.032 |  | 0.001 | -0.001, 0.003 | 0.512 |  | -0.001 | -0.011, 0.010 | 0.894 | Chi-square value: 78.0 (DF: 55, 0.022), CFI: 0.942, RMSEA: 0.029 |
|  | Sex | 0.000 | -0.139, 0.139 | 0.997 |  | 0.012 | -0.026, 0.049 | 0.544 |  | 0.055 | -0.088, 0.199 | 0.450 |  |
|  | Statin | 0.026 | -0.155, 0.208 | 0.776 |  | -0.038 | -0.080, 0.003 | 0.071 |  | -- | -- | -- |  |

Pre_slope means the slope before starting to use statins (for statin users) or the slope from the first observation to the last observation (nonstatin users); Post_slope means the slope after starting to use statins until the last observation (only for statin users). The Bonferroni adjusted significance level was set to 0.013 (0.05/4). TC: total cholesterol, LDL: low-density lipoprotein cholesterol; TG: total triglyceride; CI: confidence interval; DF: degree of freedom; CFI: comparative fit index; RMSEA: root-mean-square error of approximation.

Supplementary table 8: Sensitivity analysis of piecewise linear-linear growth curve models of latent intercept and slopes of longitudinal trends of DNA methylation on candidate CpGs by excluding samples with TG levels great than 400 mg/dL.

| Trait | Regressors | Intercept | | |  | Pre_slope | | |  | Post_slope | | | Model fit indices |
| --- | --- | --- | --- | --- | --- | --- | --- | --- | --- | --- | --- | --- | --- |
|  |  | Estimate | 95% CI | P value |  | Estimate | 95% CI | P value |  | Estimate | 95% CI | P value |  |
| cg17901584 | Age | 0.008 | -0.001, 0.016 | 0.088 |  | 0.000 | -0.004, 0.004 | 0.975 |  | -0.001 | -0.017, 0.014 | 0.851 | Chi-square value: 43.8 (DF: 32, P=0.079), CFI: 0.912, RMSEA: 0.027 |
|  | Sex | -0.227 | -0.392, -0.061 | 0.007 |  | -0.086 | -0.151, -0.022 | 0.009 |  | 0.284 | 0.022, 0.546 | 0.033 |  |
|  | Statin | -0.273 | -0.511, -0.036 | 0.024 |  | -0.065 | -0.171, 0.042 | 0.232 |  | -- | -- | -- |  |
| cg27243685 | Age | -0.005 | -0.008, -0.001 | 0.014 |  | -0.001 | -0.003, 0.002 | 0.541 |  | -0.005 | -0.019, 0.009 | 0.483 | Chi-square value: 37.4 (DF: 22, P=0.021), CFI: 0.779, RMSEA: 0.037 |
|  | Sex | -0.125 | -0.200, -0.050 | 0.001 |  | -0.030 | -0.076, 0.015 | 0.193 |  | -0.015 | -0.179, 0.148 | 0.854 |  |
|  | Statin | 0.147 | 0.065, 0.230 | <0.001 |  | 0.087 | 0.034, 0.140 | 0.001 |  | -- | -- | -- |  |

Pre_slope means the slope in the phase from the first observation of not using statin until the observation of starting to use statin (for statin users) or the slope from the first observation to the last observation of repeated measurements (nonstatin users); Post_slope means the slope in the phase from the observation of starting to use statin until the last observation of repeated measurements (only for statin users). The Bonferroni adjusted significance level was set to 0.017 (0.05/3). CI: confidence interval; DF: degree of freedom; CFI: comparative fit index; RMSEA: root-mean-square error of approximation; TG: total triglyceride. Results for cg10177197 were not shown because the model did not converge for it.

Supplementary table 9: Sensitivity analysis of cross-lagged effect between blood lipids and DNA methylation on candidate CpGs over adjacent IPTs from bivariate autoregressive latent trajectory model with structured residuals by excluding samples with TG levels greater than 400 mg/dL.

| Dependent variable | Independent variable | Statin users | | |  | Non-statin users | | |
| --- | --- | --- | --- | --- | --- | --- | --- | --- |
|  |  | Estimate | 95% CI | P value |  | Estimate | 95% CI | P value |
| cg10177197.IPT5 | TC.IPT3 | -0.038 | -0.255, 0.179 | 0.731 |  | 0.033 | -0.089, 0.154 | 0.596 |
| cg10177197.IPT6 | TC.IPT5 | -0.096 | -0.223, 0.032 | 0.142 |  | 0.105 | -0.109, 0.320 | 0.336 |
| cg10177197.IPT8 | TC.IPT6 | -0.015 | -0.249, 0.220 | 0.902 |  | 0.047 | -0.374, 0.468 | 0.826 |
| cg10177197.IPT9 | TC.IPT8 | -0.243 | -0.530, 0.043 | 0.096 |  | 0.006 | -0.192, 0.203 | 0.955 |
| TC.IPT5 | cg10177197.IPT3 | -0.075 | -1.188, 1.039 | 0.895 |  | -0.010 | -1.144, 1.124 | 0.986 |
| TC.IPT6 | cg10177197.IPT5 | -1.368 | -2.897, 0.161 | 0.080 |  | -0.125 | -0.455, 0.206 | 0.459 |
| TC.IPT8 | cg10177197.IPT6 | 0.402 | -0.211, 1.015 | 0.199 |  | 0.040 | -0.619, 0.700 | 0.905 |
| TC.IPT9 | cg10177197.IPT8 | -0.324 | -1.222, 0.574 | 0.479 |  | -0.178 | -0.556, 0.201 | 0.358 |
| cg17901584.IPT5 | TC.IPT3 | -0.703 | -2.363, 0.958 | 0.407 |  | -0.002 | -0.277, 0.274 | 0.991 |
| cg17901584.IPT6 | TC.IPT5 | -0.311 | -0.649, 0.027 | 0.072 |  | 0.032 | -0.419, 0.483 | 0.888 |
| cg17901584.IPT8 | TC.IPT6 | -0.067 | -0.347, 0.214 | 0.642 |  | 0.037 | -0.464, 0.537 | 0.886 |
| cg17901584.IPT9 | TC.IPT8 | -0.184 | -0.444, 0.075 | 0.164 |  | 0.360 | -0.079, 0.800 | 0.108 |
| TC.IPT5 | cg17901584.IPT3 | 0.072 | -0.218, 0.361 | 0.627 |  | 0.301 | -0.080, 0.682 | 0.121 |
| TC.IPT6 | cg17901584.IPT5 | -0.204 | -0.584, 0.177 | 0.295 |  | 0.178 | -0.012, 0.367 | 0.067 |
| TC.IPT8 | cg17901584.IPT6 | -0.081 | -0.549, 0.387 | 0.734 |  | -0.228 | -0.539, 0.084 | 0.153 |
| TC.IPT9 | cg17901584.IPT8 | -0.114 | -0.435, 0.207 | 0.485 |  | -0.081 | -0.255, 0.093 | 0.362 |
| cg27243685.IPT5 | TC.IPT3 | -0.054 | -0.320, 0.213 | 0.694 |  | -0.078 | -0.245, 0.089 | 0.362 |
| cg27243685.IPT6 | TC.IPT5 | -0.033 | -1.236, 1.169 | 0.957 |  | 0.023 | -0.169, 0.215 | 0.811 |
| cg27243685.IPT8 | TC.IPT6 | 0.007 | -0.200, 0.215 | 0.944 |  | 0.013 | -0.145, 0.170 | 0.872 |
| cg27243685.IPT9 | TC.IPT8 | 0.056 | -0.239, 0.350 | 0.711 |  | -0.176 | -0.440, 0.087 | 0.189 |
| TC.IPT5 | cg27243685.IPT3 | -0.572 | -2.424, 1.279 | 0.545 |  | 0.401 | -0.248, 1.049 | 0.226 |
| TC.IPT6 | cg27243685.IPT5 | -1.156 | -5.067, 2.754 | 0.562 |  | 0.117 | -0.209, 0.442 | 0.483 |
| TC.IPT8 | cg27243685.IPT6 | -0.433 | -2.853, 1.988 | 0.726 |  | 0.242 | -0.402, 0.885 | 0.462 |
| TC.IPT9 | cg27243685.IPT8 | 0.207 | -1.740, 2.155 | 0.835 |  | -0.087 | -0.427, 0.253 | 0.616 |
| cg10177197.IPT6 | LDL.IPT5 | -0.098 | -0.299, 0.104 | 0.341 |  | 0.275 | -0.372, 0.922 | 0.405 |
| cg10177197.IPT8 | LDL.IPT6 | -0.099 | -0.327, 0.129 | 0.394 |  | 0.041 | -0.341, 0.423 | 0.833 |
| cg10177197.IPT9 | LDL.IPT8 | -0.215 | -0.456, 0.027 | 0.081 |  | 0.019 | -0.431, 0.469 | 0.933 |
| LDL.IPT6 | cg10177197.IPT5 | -1.228 | -3.601, 1.145 | 0.310 |  | 0.168 | -0.718, 1.053 | 0.710 |
| LDL.IPT8 | cg10177197.IPT6 | 0.325 | -0.794, 1.445 | 0.569 |  | 0.082 | -0.822, 0.986 | 0.860 |
| LDL.IPT9 | cg10177197.IPT8 | -0.555 | -1.691, 0.580 | 0.338 |  | -0.079 | -0.586, 0.428 | 0.760 |
| cg17901584.IPT6 | LDL.IPT5 | -0.134 | -0.555, 0.287 | 0.533 |  | 0.347 | -0.603, 1.297 | 0.474 |
| cg17901584.IPT8 | LDL.IPT6 | -0.080 | -0.386, 0.227 | 0.611 |  | 0.123 | -0.659, 0.905 | 0.758 |
| cg17901584.IPT9 | LDL.IPT8 | -0.070 | -0.467, 0.327 | 0.729 |  | 0.301 | -0.204, 0.806 | 0.243 |
| LDL.IPT6 | cg17901584.IPT5 | -0.070 | -0.734, 0.595 | 0.837 |  | 0.231 | -0.119, 0.581 | 0.195 |
| LDL.IPT8 | cg17901584.IPT6 | 0.009 | -0.542, 0.560 | 0.974 |  | -0.172 | -0.480, 0.137 | 0.275 |
| LDL.IPT9 | cg17901584.IPT8 | -0.073 | -0.636, 0.491 | 0.800 |  | -0.007 | -0.258, 0.243 | 0.954 |
| cg27243685.IPT6 | LDL.IPT5 | -0.058 | -0.281, 0.166 | 0.613 |  | 0.523 | -0.495, 1.540 | 0.314 |
| cg27243685.IPT8 | LDL.IPT6 | -0.016 | -0.159, 0.127 | 0.826 |  | 0.191 | -0.261, 0.644 | 0.408 |
| cg27243685.IPT9 | LDL.IPT8 | 0.158 | -0.060, 0.377 | 0.155 |  | 0.040 | -0.371, 0.451 | 0.850 |
| LDL.IPT6 | cg27243685.IPT5 | -0.658 | -2.029, 0.713 | 0.347 |  | 0.986 | -0.444, 2.415 | 0.177 |
| LDL.IPT8 | cg27243685.IPT6 | -0.258 | -2.617, 2.101 | 0.830 |  | 0.227 | -0.614, 1.067 | 0.597 |
| LDL.IPT9 | cg27243685.IPT8 | 0.357 | -1.538, 2.253 | 0.712 |  | 0.235 | -0.173, 0.643 | 0.258 |
| cg10177197.IPT6 | HDL.IPT5 | -0.953 | -2.598, 0.691 | 0.256 |  | 0.186 | -0.342, 0.714 | 0.490 |
| cg10177197.IPT8 | HDL.IPT6 | -0.320 | -1.453, 0.813 | 0.580 |  | 0.029 | -0.647, 0.705 | 0.933 |
| cg10177197.IPT9 | HDL.IPT8 | 0.433 | -1.120, 1.985 | 0.585 |  | 0.344 | -0.172, 0.860 | 0.191 |
| HDL.IPT6 | cg10177197.IPT5 | -0.210 | -0.537, 0.116 | 0.206 |  | 0.050 | -0.069, 0.168 | 0.412 |
| HDL.IPT8 | cg10177197.IPT6 | -0.233 | -0.451, -0.015 | 0.036 |  | 0.168 | -0.048, 0.384 | 0.129 |
| HDL.IPT9 | cg10177197.IPT8 | -0.046 | -0.376, 0.283 | 0.783 |  | 0.106 | -0.064, 0.275 | 0.222 |
| cg17901584.IPT6 | HDL.IPT5 | -0.164 | -4.083, 3.756 | 0.935 |  | 6.311 | -23.703, 36.324 | 0.680 |
| cg17901584.IPT8 | HDL.IPT6 | -1.367 | -3.569, 0.835 | 0.224 |  | 0.325 | -0.873, 1.523 | 0.595 |
| cg17901584.IPT9 | HDL.IPT8 | -5.578 | -14.751, 3.594 | 0.233 |  | 0.644 | -1.646, 2.934 | 0.581 |
| HDL.IPT6 | cg17901584.IPT5 | -0.235 | -0.549, 0.080 | 0.144 |  | 0.722 | -3.959, 5.403 | 0.762 |
| HDL.IPT8 | cg17901584.IPT6 | -0.056 | -0.162, 0.050 | 0.298 |  | -0.025 | -0.074, 0.024 | 0.322 |
| HDL.IPT9 | cg17901584.IPT8 | -0.553 | -1.630, 0.524 | 0.314 |  | 0.064 | -0.054, 0.182 | 0.288 |
| cg27243685.IPT6 | HDL.IPT5 | 0.470 | -1.114, 2.054 | 0.561 |  | -2.598 | -27.245, 22.050 | 0.836 |
| cg27243685.IPT8 | HDL.IPT6 | 0.343 | -0.634, 1.320 | 0.492 |  | 0.080 | -0.562, 0.722 | 0.808 |
| cg27243685.IPT9 | HDL.IPT8 | 0.735 | -0.948, 2.418 | 0.392 |  | -0.199 | -1.427, 1.030 | 0.751 |
| HDL.IPT6 | cg27243685.IPT5 | 0.017 | -1.221, 1.254 | 0.979 |  | -0.649 | -4.096, 2.799 | 0.712 |
| HDL.IPT8 | cg27243685.IPT6 | 0.191 | -0.123, 0.506 | 0.233 |  | -0.006 | -0.172, 0.160 | 0.940 |
| HDL.IPT9 | cg27243685.IPT8 | 0.244 | -0.365, 0.852 | 0.432 |  | -0.005 | -0.364, 0.354 | 0.978 |
| **cg10177197.IPT5** | **TG.IPT3** | 0.059 | -0.384, 0.502 | 0.793 |  | **-0.427** | **-0.702, -0.152** | **0.002** |
| cg10177197.IPT6 | TG.IPT5 | 0.163 | -0.186, 0.513 | 0.359 |  | -0.061 | -0.307, 0.184 | 0.625 |
| cg10177197.IPT8 | TG.IPT6 | -0.133 | -0.427, 0.162 | 0.377 |  | 0.103 | -0.199, 0.405 | 0.504 |
| cg10177197.IPT9 | TG.IPT8 | -0.100 | -0.480, 0.281 | 0.607 |  | -0.043 | -0.288, 0.201 | 0.727 |
| TG.IPT5 | cg10177197.IPT3 | 0.216 | -0.235, 0.668 | 0.348 |  | -0.563 | -1.324, 0.198 | 0.147 |
| TG.IPT6 | cg10177197.IPT5 | -0.469 | -1.272, 0.335 | 0.253 |  | -0.107 | -0.889, 0.676 | 0.789 |
| TG.IPT8 | cg10177197.IPT6 | 0.007 | -0.454, 0.468 | 0.975 |  | -0.147 | -0.659, 0.366 | 0.575 |
| TG.IPT9 | cg10177197.IPT8 | -0.231 | -0.691, 0.228 | 0.324 |  | -0.288 | -0.515, -0.061 | 0.013 |
| cg17901584.IPT5 | TG.IPT3 | 1.252 | -6.696, 9.200 | 0.758 |  | -0.214 | -0.627, 0.199 | 0.309 |
| cg17901584.IPT6 | TG.IPT5 | -0.446 | -1.430, 0.538 | 0.374 |  | 0.083 | -0.306, 0.473 | 0.675 |
| cg17901584.IPT8 | TG.IPT6 | -0.346 | -1.048, 0.355 | 0.333 |  | 0.298 | -0.413, 1.010 | 0.412 |
| cg17901584.IPT9 | TG.IPT8 | 0.003 | -0.442, 0.448 | 0.989 |  | 0.267 | -0.186, 0.721 | 0.248 |
| TG.IPT5 | cg17901584.IPT3 | -0.112 | -0.661, 0.436 | 0.688 |  | -0.125 | -0.308, 0.058 | 0.182 |
| TG.IPT6 | cg17901584.IPT5 | 0.083 | -0.328, 0.495 | 0.691 |  | 0.036 | -0.132, 0.204 | 0.675 |
| TG.IPT8 | cg17901584.IPT6 | 0.018 | -0.263, 0.298 | 0.902 |  | -0.024 | -0.207, 0.158 | 0.795 |
| TG.IPT9 | cg17901584.IPT8 | -0.081 | -0.223, 0.061 | 0.265 |  | -0.098 | -0.228, 0.032 | 0.138 |
| cg27243685.IPT5 | TG.IPT3 | 0.089 | -0.159, 0.337 | 0.482 |  | 0.040 | -0.881, 0.961 | 0.932 |
| **cg27243685.IPT6** | **TG.IPT5** | **0.369** | **0.147, 0.591** | **0.001** |  | -0.077 | -0.617, 0.464 | 0.780 |
| cg27243685.IPT8 | TG.IPT6 | -0.115 | -0.482, 0.252 | 0.539 |  | -0.052 | -0.617, 0.513 | 0.857 |
| cg27243685.IPT9 | TG.IPT8 | -0.050 | -0.409, 0.309 | 0.784 |  | -0.013 | -0.492, 0.465 | 0.956 |
| TG.IPT5 | cg27243685.IPT3 | 0.447 | -0.172, 1.066 | 0.157 |  | 0.007 | -0.844, 0.858 | 0.987 |
| TG.IPT6 | cg27243685.IPT5 | -0.861 | -1.767, 0.044 | 0.062 |  | 0.193 | -1.224, 1.609 | 0.790 |
| TG.IPT8 | cg27243685.IPT6 | 0.232 | -0.448, 0.912 | 0.504 |  | -0.197 | -0.833, 0.439 | 0.544 |
| TG.IPT9 | cg27243685.IPT8 | -0.272 | -0.813, 0.269 | 0.325 |  | -0.004 | -0.653, 0.645 | 0.990 |

Baseline age, sex and smoking were adjusted as covariates. TC: total cholesterol, LDL: low-density lipoprotein cholesterol; HDL: high-density lipoprotein cholesterol; TG: total triglyceride; CI: confidence interval; IPT: in-person testing. “-” means we did not get converged result from this bivariate autoregressive latent trajectory model with structured residuals.

Supplementary Table 10: Associations between statin use and blood lipids and DNA methylation on candidate CpGs from mixed effect model

| Dependent variables | Estimate | 95% CI | P value |
| --- | --- | --- | --- |
| TC | -0.460 | -0.672, -0.246 | 2.76E-05 |
| LDL | -0.565 | -0.771, -0.358 | 1.23E-07 |
| HDL | 0.067 | -0.017, 0.151 | 0.118 |
| TG | 0.097 | -0.067, 0.266 | 0.257 |
| cg10177197 | 0.004 | -0.003, 0.010 | 0.260 |
| cg17901584 | -0.013 | -0.028, 0.002 | 0.091 |
| cg27243685 | 0.009 | 0.001, 0.016 | 0.017 |

Age, sex and smoking were adjusted for as covariates, and additionally TC for the CpG models. TC was included as a covariate to be comparable to previous work by C Ochoa-Rosales et al (2020). TC: total cholesterol, LDL: low-density lipoprotein cholesterol; HDL: high-density lipoprotein cholesterol; TG: total triglyceride; CI: confidence interval. The Bonferroni adjusted significance level was set to 0.013 (0.05/4) and 0.017 (0.05/3) for lipids and CpGs, respectively.
