# Supplementary figures and images for: Dynamic patterns of blood lipids and DNA methylation in response to statin therapy
Source: Clin Epigenetics. 2022 Nov 28;14:153. doi: 10.1186/s13148-022-01375-8 (PMC9706978; doi:10.1186/s13148-022-01375-8)

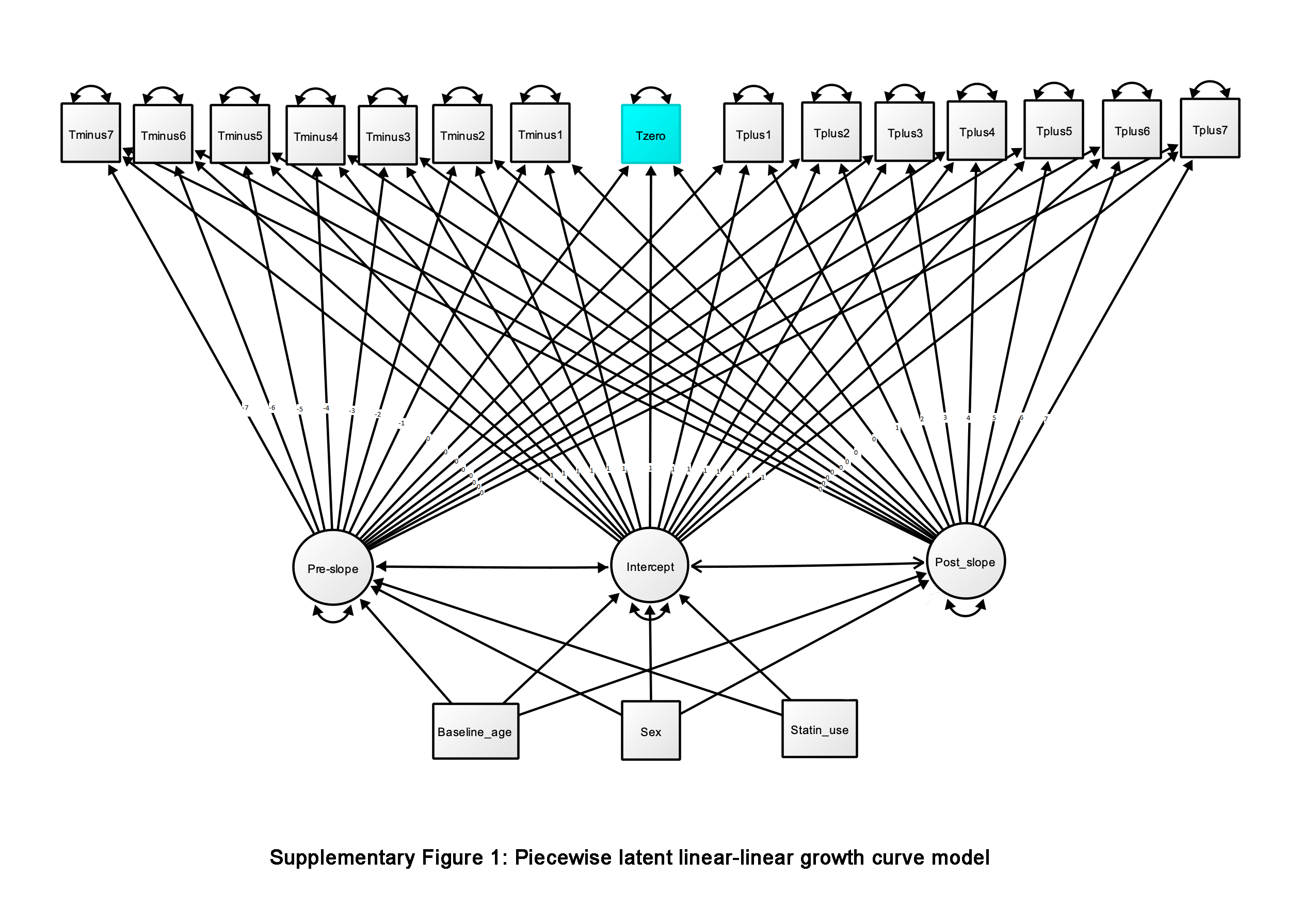

Supplement: Supplementary file 1 — Additional file 1. Figure S1: Piecewise latent linear-linear growth curve model. This is a schematic diagram depicting the piecewise latent linear-linear growth curve model. The observed variables are shown as rectangles and latent variables as circles; the double-headed arrows represent variance or covariance of variables and single-headed arrows represent regression effects with the variable at the tail of the arrow having causal effect on the variable at the head. The regression effects are also called paths, directional effects and factor loadings, with the latter specifying the regression coefficients linking latent variables and observed variables. Tzero: the lipids (or DNA methylation) level at the time (denoted as in-person testing (IPT) in our study) of start to use statin. Tminus1-Tminus7: the lipids (or DNA methylation) level before statin treatment, and the suffix numbers of Tminus are determined by how many folds of the time interval deviating to Tzero. Tplus1-Tplus7: the lipids (or DNA methylation) level after statin treatment, and the suffix numbers have the same definition as that in Tminus. Intercept: the individual lipid (or DNA methylation) level at Tzero. Pre-slope: the changing rate of lipids (or DNA methylation) over time before the changing point for each individual. Post-slope: the changing rate of lipids (or DNA methylation) over time after the changing point for each individual. The factor loadings from the latent intercept to Tminus and Tplus variables are all set to 1. The factor loadings from latent “Pre_slope” are set as -7 to -1 for Tminus7 to Tminus1 and were equal to 0 for all Tplus variables. The factor loadings from the “Post_slope” to Tminus variables are all set as 0 but are set as 1 to 7 for Tplus1 to Tplus7, respectively. Baseline age, sex, and statin use are included as time-independent covariates as we assumed that these variables would have associations with latent intercept and slopes. Figure S2: Bivariate autoregressive la [file 13148_2022_1375_MOESM1_ESM.zip › supplementary file-revised version2/supple fig 1-pw model.png]

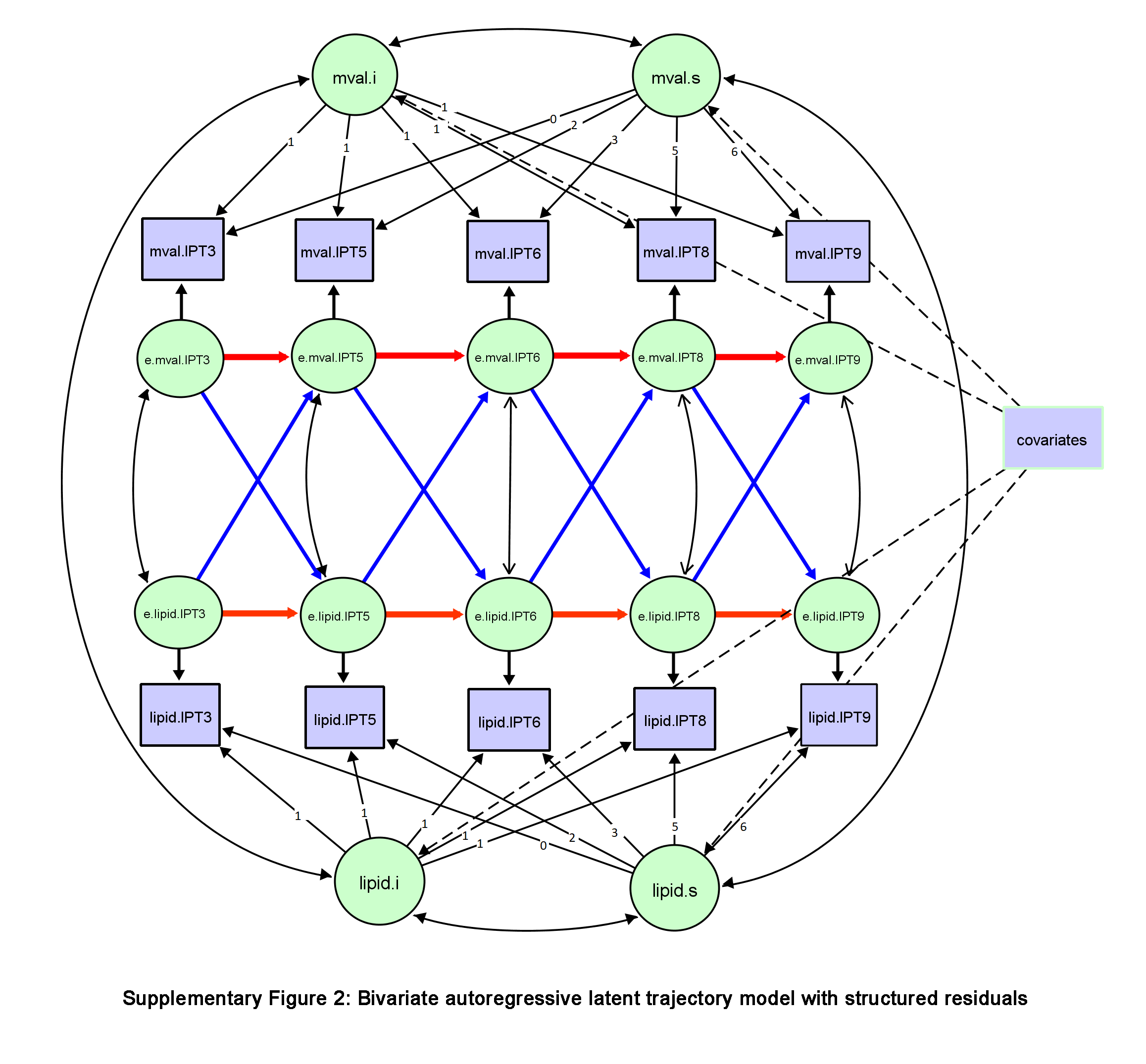

Supplement: Supplementary file 1 — Additional file 1. Figure S1: Piecewise latent linear-linear growth curve model. This is a schematic diagram depicting the piecewise latent linear-linear growth curve model. The observed variables are shown as rectangles and latent variables as circles; the double-headed arrows represent variance or covariance of variables and single-headed arrows represent regression effects with the variable at the tail of the arrow having causal effect on the variable at the head. The regression effects are also called paths, directional effects and factor loadings, with the latter specifying the regression coefficients linking latent variables and observed variables. Tzero: the lipids (or DNA methylation) level at the time (denoted as in-person testing (IPT) in our study) of start to use statin. Tminus1-Tminus7: the lipids (or DNA methylation) level before statin treatment, and the suffix numbers of Tminus are determined by how many folds of the time interval deviating to Tzero. Tplus1-Tplus7: the lipids (or DNA methylation) level after statin treatment, and the suffix numbers have the same definition as that in Tminus. Intercept: the individual lipid (or DNA methylation) level at Tzero. Pre-slope: the changing rate of lipids (or DNA methylation) over time before the changing point for each individual. Post-slope: the changing rate of lipids (or DNA methylation) over time after the changing point for each individual. The factor loadings from the latent intercept to Tminus and Tplus variables are all set to 1. The factor loadings from latent “Pre_slope” are set as -7 to -1 for Tminus7 to Tminus1 and were equal to 0 for all Tplus variables. The factor loadings from the “Post_slope” to Tminus variables are all set as 0 but are set as 1 to 7 for Tplus1 to Tplus7, respectively. Baseline age, sex, and statin use are included as time-independent covariates as we assumed that these variables would have associations with latent intercept and slopes. Figure S2: Bivariate autoregressive la [file 13148_2022_1375_MOESM1_ESM.zip › supplementary file-revised version2/supple figure 2 ALT-SR for statin manuscript.png]

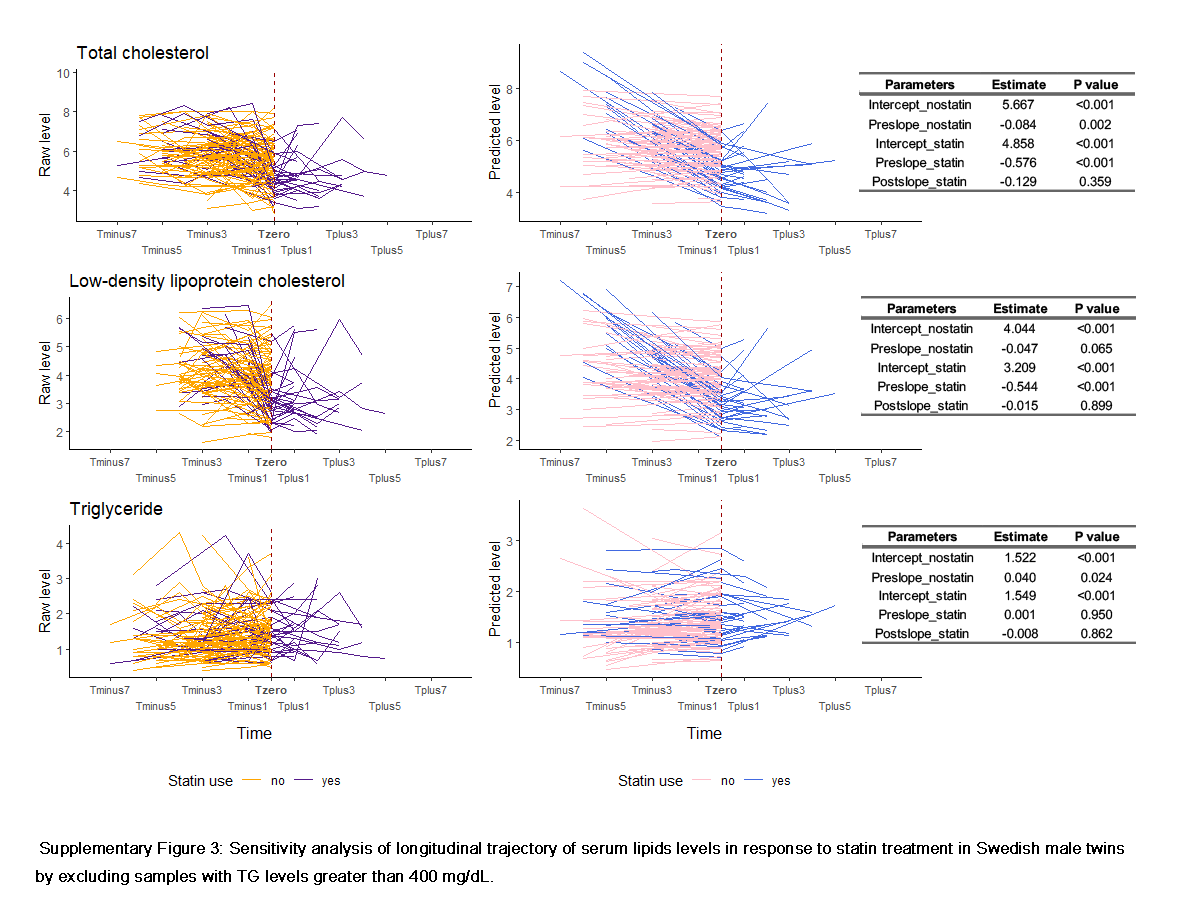

Supplement: Supplementary file 1 — Additional file 1. Figure S1: Piecewise latent linear-linear growth curve model. This is a schematic diagram depicting the piecewise latent linear-linear growth curve model. The observed variables are shown as rectangles and latent variables as circles; the double-headed arrows represent variance or covariance of variables and single-headed arrows represent regression effects with the variable at the tail of the arrow having causal effect on the variable at the head. The regression effects are also called paths, directional effects and factor loadings, with the latter specifying the regression coefficients linking latent variables and observed variables. Tzero: the lipids (or DNA methylation) level at the time (denoted as in-person testing (IPT) in our study) of start to use statin. Tminus1-Tminus7: the lipids (or DNA methylation) level before statin treatment, and the suffix numbers of Tminus are determined by how many folds of the time interval deviating to Tzero. Tplus1-Tplus7: the lipids (or DNA methylation) level after statin treatment, and the suffix numbers have the same definition as that in Tminus. Intercept: the individual lipid (or DNA methylation) level at Tzero. Pre-slope: the changing rate of lipids (or DNA methylation) over time before the changing point for each individual. Post-slope: the changing rate of lipids (or DNA methylation) over time after the changing point for each individual. The factor loadings from the latent intercept to Tminus and Tplus variables are all set to 1. The factor loadings from latent “Pre_slope” are set as -7 to -1 for Tminus7 to Tminus1 and were equal to 0 for all Tplus variables. The factor loadings from the “Post_slope” to Tminus variables are all set as 0 but are set as 1 to 7 for Tplus1 to Tplus7, respectively. Baseline age, sex, and statin use are included as time-independent covariates as we assumed that these variables would have associations with latent intercept and slopes. Figure S2: Bivariate autoregressive la [file 13148_2022_1375_MOESM1_ESM.zip › supplementary file-revised version2/supple figure 3 new.png]

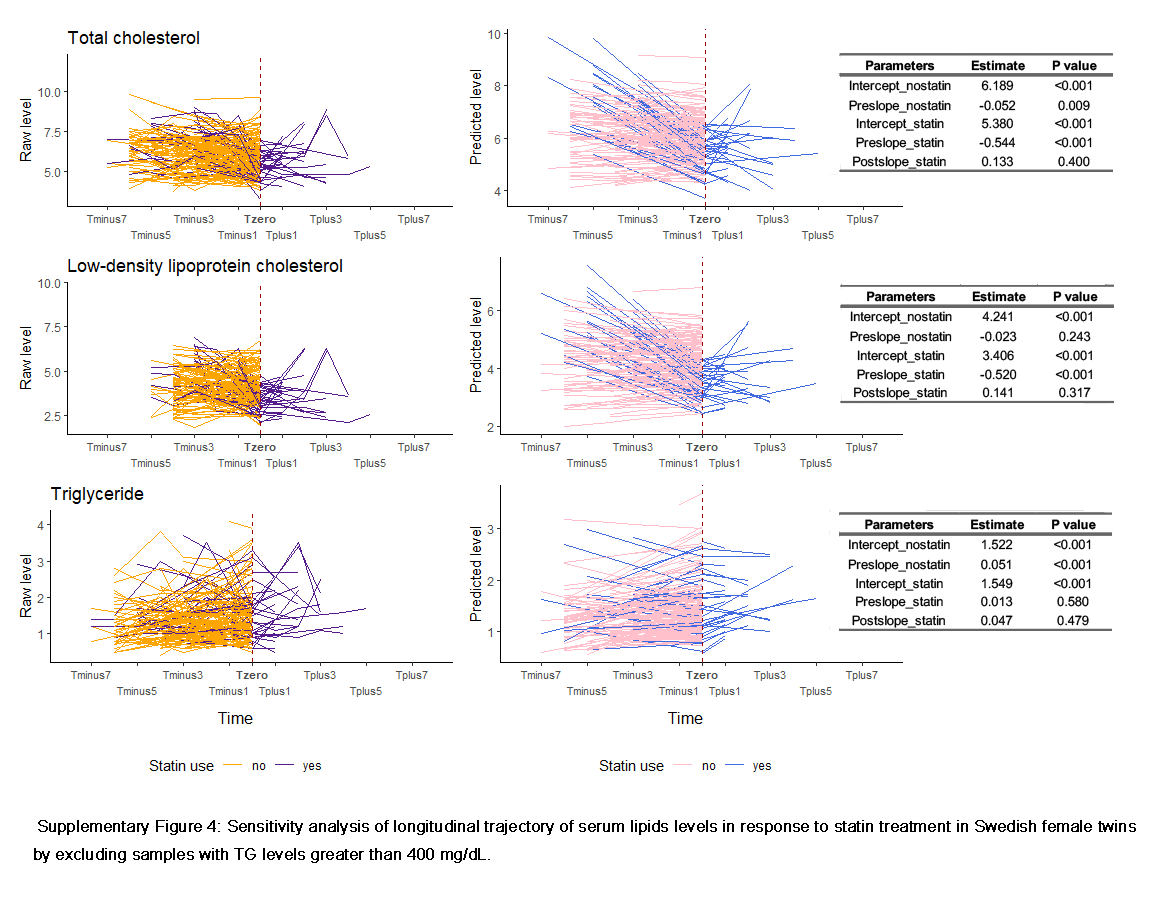

Supplement: Supplementary file 1 — Additional file 1. Figure S1: Piecewise latent linear-linear growth curve model. This is a schematic diagram depicting the piecewise latent linear-linear growth curve model. The observed variables are shown as rectangles and latent variables as circles; the double-headed arrows represent variance or covariance of variables and single-headed arrows represent regression effects with the variable at the tail of the arrow having causal effect on the variable at the head. The regression effects are also called paths, directional effects and factor loadings, with the latter specifying the regression coefficients linking latent variables and observed variables. Tzero: the lipids (or DNA methylation) level at the time (denoted as in-person testing (IPT) in our study) of start to use statin. Tminus1-Tminus7: the lipids (or DNA methylation) level before statin treatment, and the suffix numbers of Tminus are determined by how many folds of the time interval deviating to Tzero. Tplus1-Tplus7: the lipids (or DNA methylation) level after statin treatment, and the suffix numbers have the same definition as that in Tminus. Intercept: the individual lipid (or DNA methylation) level at Tzero. Pre-slope: the changing rate of lipids (or DNA methylation) over time before the changing point for each individual. Post-slope: the changing rate of lipids (or DNA methylation) over time after the changing point for each individual. The factor loadings from the latent intercept to Tminus and Tplus variables are all set to 1. The factor loadings from latent “Pre_slope” are set as -7 to -1 for Tminus7 to Tminus1 and were equal to 0 for all Tplus variables. The factor loadings from the “Post_slope” to Tminus variables are all set as 0 but are set as 1 to 7 for Tplus1 to Tplus7, respectively. Baseline age, sex, and statin use are included as time-independent covariates as we assumed that these variables would have associations with latent intercept and slopes. Figure S2: Bivariate autoregressive la [file 13148_2022_1375_MOESM1_ESM.zip › supplementary file-revised version2/supple figure 4 new.png]

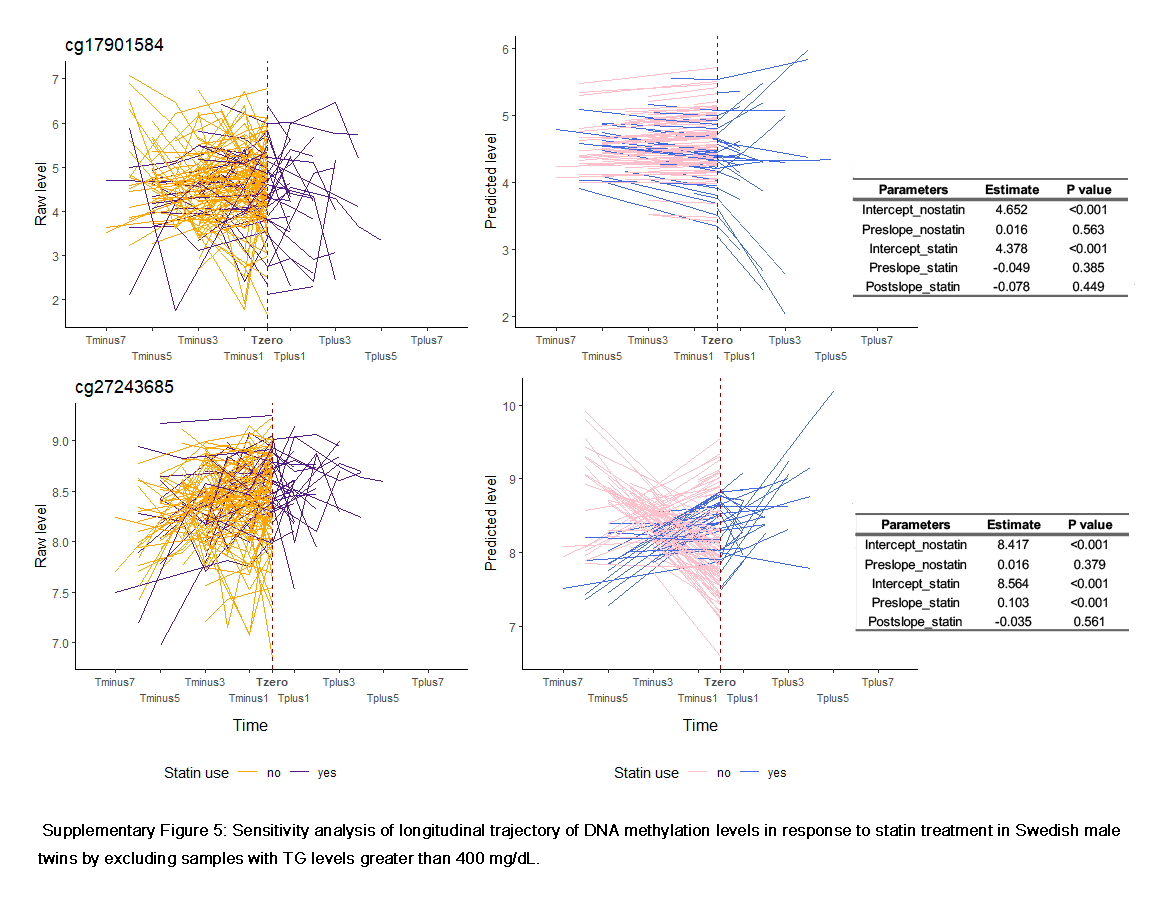

Supplement: Supplementary file 1 — Additional file 1. Figure S1: Piecewise latent linear-linear growth curve model. This is a schematic diagram depicting the piecewise latent linear-linear growth curve model. The observed variables are shown as rectangles and latent variables as circles; the double-headed arrows represent variance or covariance of variables and single-headed arrows represent regression effects with the variable at the tail of the arrow having causal effect on the variable at the head. The regression effects are also called paths, directional effects and factor loadings, with the latter specifying the regression coefficients linking latent variables and observed variables. Tzero: the lipids (or DNA methylation) level at the time (denoted as in-person testing (IPT) in our study) of start to use statin. Tminus1-Tminus7: the lipids (or DNA methylation) level before statin treatment, and the suffix numbers of Tminus are determined by how many folds of the time interval deviating to Tzero. Tplus1-Tplus7: the lipids (or DNA methylation) level after statin treatment, and the suffix numbers have the same definition as that in Tminus. Intercept: the individual lipid (or DNA methylation) level at Tzero. Pre-slope: the changing rate of lipids (or DNA methylation) over time before the changing point for each individual. Post-slope: the changing rate of lipids (or DNA methylation) over time after the changing point for each individual. The factor loadings from the latent intercept to Tminus and Tplus variables are all set to 1. The factor loadings from latent “Pre_slope” are set as -7 to -1 for Tminus7 to Tminus1 and were equal to 0 for all Tplus variables. The factor loadings from the “Post_slope” to Tminus variables are all set as 0 but are set as 1 to 7 for Tplus1 to Tplus7, respectively. Baseline age, sex, and statin use are included as time-independent covariates as we assumed that these variables would have associations with latent intercept and slopes. Figure S2: Bivariate autoregressive la [file 13148_2022_1375_MOESM1_ESM.zip › supplementary file-revised version2/supple figure 5 new.png]

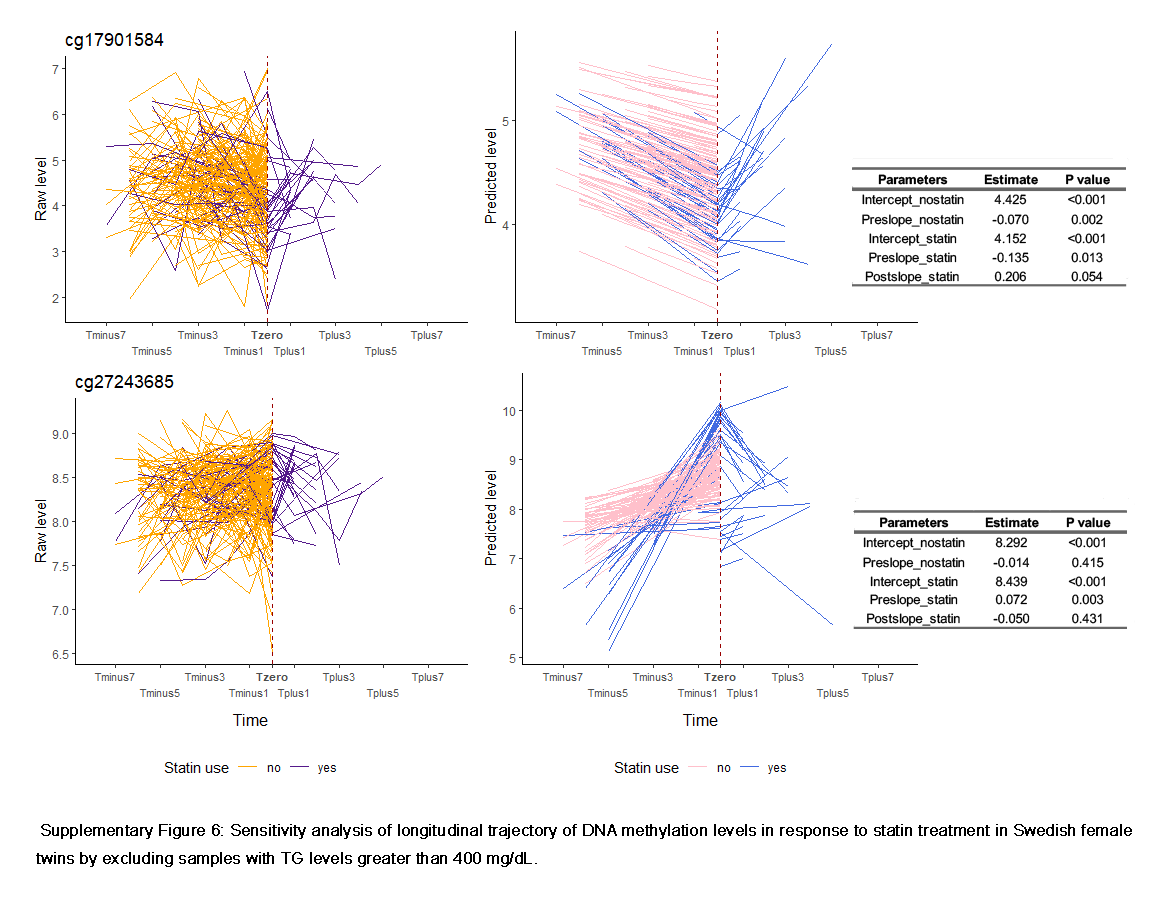

Supplement: Supplementary file 1 — Additional file 1. Figure S1: Piecewise latent linear-linear growth curve model. This is a schematic diagram depicting the piecewise latent linear-linear growth curve model. The observed variables are shown as rectangles and latent variables as circles; the double-headed arrows represent variance or covariance of variables and single-headed arrows represent regression effects with the variable at the tail of the arrow having causal effect on the variable at the head. The regression effects are also called paths, directional effects and factor loadings, with the latter specifying the regression coefficients linking latent variables and observed variables. Tzero: the lipids (or DNA methylation) level at the time (denoted as in-person testing (IPT) in our study) of start to use statin. Tminus1-Tminus7: the lipids (or DNA methylation) level before statin treatment, and the suffix numbers of Tminus are determined by how many folds of the time interval deviating to Tzero. Tplus1-Tplus7: the lipids (or DNA methylation) level after statin treatment, and the suffix numbers have the same definition as that in Tminus. Intercept: the individual lipid (or DNA methylation) level at Tzero. Pre-slope: the changing rate of lipids (or DNA methylation) over time before the changing point for each individual. Post-slope: the changing rate of lipids (or DNA methylation) over time after the changing point for each individual. The factor loadings from the latent intercept to Tminus and Tplus variables are all set to 1. The factor loadings from latent “Pre_slope” are set as -7 to -1 for Tminus7 to Tminus1 and were equal to 0 for all Tplus variables. The factor loadings from the “Post_slope” to Tminus variables are all set as 0 but are set as 1 to 7 for Tplus1 to Tplus7, respectively. Baseline age, sex, and statin use are included as time-independent covariates as we assumed that these variables would have associations with latent intercept and slopes. Figure S2: Bivariate autoregressive la [file 13148_2022_1375_MOESM1_ESM.zip › supplementary file-revised version2/supple figure 6 new.png]
